# Metabolic Effects of *n*-3 PUFA as Phospholipids Are Superior to Triglycerides in Mice Fed a High-Fat Diet: Possible Role of Endocannabinoids

**DOI:** 10.1371/journal.pone.0038834

**Published:** 2012-06-11

**Authors:** Martin Rossmeisl, Zuzana Macek Jilkova, Ondrej Kuda, Tomas Jelenik, Dasa Medrikova, Barbora Stankova, Björn Kristinsson, Gudmundur G. Haraldsson, Harald Svensen, Iren Stoknes, Peter Sjövall, Ylva Magnusson, Michiel G. J. Balvers, Kitty C. M. Verhoeckx, Eva Tvrzicka, Morten Bryhn, Jan Kopecky

**Affiliations:** 1 Department of Adipose Tissue Biology, Institute of Physiology Academy of Sciences of the Czech Republic v.v.i., Prague, Czech Republic; 2 4th Department of Internal Medicine, 1st Faculty of Medicine, Charles University, Prague, Czech Republic; 3 Science Institute, University of Iceland, Reykjavik, Iceland; 4 EPAX AS, Aalesund, Norway; 5 SP Technical Research Institute of Sweden, Borås, Sweden; 6 Wallenberg Laboratory, The Sahlgrenska Academy and University Hospital, University of Gothenburg, Gothenburg, Sweden; 7 Division of Human Nutrition, Wageningen University, Wageningen, The Netherlands; 8 Research Group Quality & Safety, TNO, Zeist, the Netherlands; 9 Silentia AS, Svelvik, Norway; Max Delbrueck Center for Molecular Medicine, Germany

## Abstract

**Background:**

*n*-3 polyunsaturated fatty acids, namely docosahexaenoic acid (DHA) and eicosapentaenoic acid (EPA), reduce the risk of cardiovascular disease and can ameliorate many of obesity-associated disorders. We hypothesised that the latter effect will be more pronounced when DHA/EPA is supplemented as phospholipids rather than as triglycerides.

**Methodology/Principal Findings:**

In a ‘prevention study’, C57BL/6J mice were fed for 9 weeks on either a corn oil-based high-fat obesogenic diet (cHF; lipids ∼35% wt/wt), or cHF-based diets in which corn oil was partially replaced by DHA/EPA, admixed either as phospholipids or triglycerides from marine fish. The reversal of obesity was studied in mice subjected to the preceding cHF-feeding for 4 months. DHA/EPA administered as phospholipids prevented glucose intolerance and tended to reduce obesity better than triglycerides. Lipemia and hepatosteatosis were suppressed more in response to dietary phospholipids, in correlation with better bioavailability of DHA and EPA, and a higher DHA accumulation in the liver, white adipose tissue (WAT), and muscle phospholipids. In dietary obese mice, both DHA/EPA concentrates prevented a further weight gain, reduced plasma lipid levels to a similar extent, and tended to improve glucose tolerance. Importantly, only the phospholipid form reduced plasma insulin and adipocyte hypertrophy, while being more effective in reducing hepatic steatosis and low-grade inflammation of WAT. These beneficial effects were correlated with changes of endocannabinoid metabolome in WAT, where phospholipids reduced 2-arachidonoylglycerol, and were more effective in increasing anti-inflammatory lipids such as *N*-docosahexaenoylethanolamine.

**Conclusions/Significance:**

Compared with triglycerides, dietary DHA/EPA administered as phospholipids are superior in preserving a healthy metabolic profile under obesogenic conditions, possibly reflecting better bioavalability and improved modulation of the endocannabinoid system activity in WAT.

## Introduction

Obesity and associated diseases such as type 2 diabetes, dyslipidemia and hypertension, i.e. components of the metabolic syndrome [1], are a major public health problem. While effective pharmacological interventions for the treatment of obesity-associated diseases require the use of multiple agents and are often associated with adverse side-effects, lifestyle modifications remain an essential component of treatment strategies. In this respect, dietary intake of marine fish oils, namely long-chain *n*-3 polyunsaturated fatty acids (**LC** ***n*** **-3 PUFA**) such as docosahexaenoic acid (**DHA**; 22∶6*n*-3) and eicosapentaenoic acid (**EPA**; 20∶5*n*-3), have been shown to exert profound hypolipidemic effects [2], [3], reducing systemic inflammation [4], [5] as well as low-grade inflammation of white adipose tissue (**WAT**; [6]–[9]) and limiting hepatosteatosis [8]–[12], resulting in a decrease in cardiovascular morbidity and the incidence of type 2 diabetes in humans [13], [14]. The cardioprotective effects have been especially pronounced in diabetic patients who have had myocardial infarction [15]. However, in spite of the fact that LC *n*-3 PUFA could help to reduce obesity in humans [16], [17] and prevent insulin resistance in rodents [8], [18], they do not reverse insulin resistance in diabetic patients [19].

Metabolic effects of LC *n*-3 PUFA largely depend on modulation of gene expression mediated by a peroxisome proliferator-activated receptor (**PPAR**)-α, -δ (-β), liver X receptor-α, hepatic nuclear factor-4, and sterol regulatory element-binding protein-1 [20], as well as on the induction of adiponectin [3], [21] and production of eicosanoids and other lipid mediators from PUFA [9], [20], [22]. Also the activity of endocannabinoid system [23], which is dysregulated in obesity [24], is modulated in response to LC *n*-3 PUFA intake. Thus, overactivity of the endocannabinoid system in tissues of genetically obese *fa*/*fa* rats [25] and mice with high-fat diet-induced obesity [11], [26] was counteracted by LC *n*-3 PUFA administered as phospholipids in krill oil. The inhibition of the endocannabinoid system activity leads to beneficial metabolic effects, reflecting multiple mechanisms of endocannabinoid action mediated by their specific receptors as well as by PPAR-α (reviewed in [23], [27]).

Compared with fish oil, containing LC *n*-3 PUFA as triglycerides, krill oil was more effective in reducing the levels of two major endogenous ligands, 2-arachidonoylglycerol (**2-AG**) and anandamide (**AEA**), containing arachidonic acid (**AA**; 20∶4*n*-6) in their backbone, in visceral adipose tissue and in the liver and heart, respectively [25]. Krill oil also improved blood lipid levels and reduced hepatic steatosis and glycemia in the obese mice [11], [26]. It is likely that supplementation of LC *n*-3 PUFA as phospholipids exerts stronger biological effects compared with the triglyceride form because (i) various phospholipid species can also act as ligands for nuclear receptors, such as PPAR-α [28], steroidogenic factor-1, and liver receptor homolog-1 [29], which are involved in the transcriptional regulation of steroidogenesis and cholesterol metabolism; and (ii) the phospholipid form has been shown to augment the bioavailability of DHA and EPA in both rodents [30]–[32] and humans [33]–[35].

In the context of animal models of metabolic syndrome, experiments focused on the modulation of tissue endocannabinoid levels by krill oil have been performed [11], [26]. However, direct comparison of the effects of the triglyceride and phospholipid forms of LC *n*-3 PUFA supplementation was only carried out in the *fa*/*fa* rats fed a chow diet [25], while a similar experiment using a model of diet-induced obesity has not been performed to date. In humans, no studies exist of the potential benefits of the phospholipid form of LC *n*-3 PUFA delivery with respect to the prevention and treatment of obesity-associated diseases.

Here, we examined the efficacy of various forms of LC *n*-3 PUFA in both the prevention and reversal of obesity and associated metabolic disorders induced by high-fat feeding in obesity-prone C57BL/6J mice. The results suggest that dietary DHA and EPA in the form of marine fish phospholipids are superior to triglycerides with respect to the preservation of glucose homeostasis and the reversal of hepatic steatosis, adipocyte hypertrophy and low-grade WAT inflammation. The higher efficacy of LC *n*-3 PUFA administered as phospholipids was associated with their better bioavailability, and with a relatively strong suppression of the levels of major endocannabinoids in WAT and plasma, suggesting that modulation of the endocannabinoid system activity contributed to their superior efficacy when compared to triglyceride form of LC *n*-3 PUFA.

## Results

### Prevention of Obesity-associated Disorders

**Figure 1:**
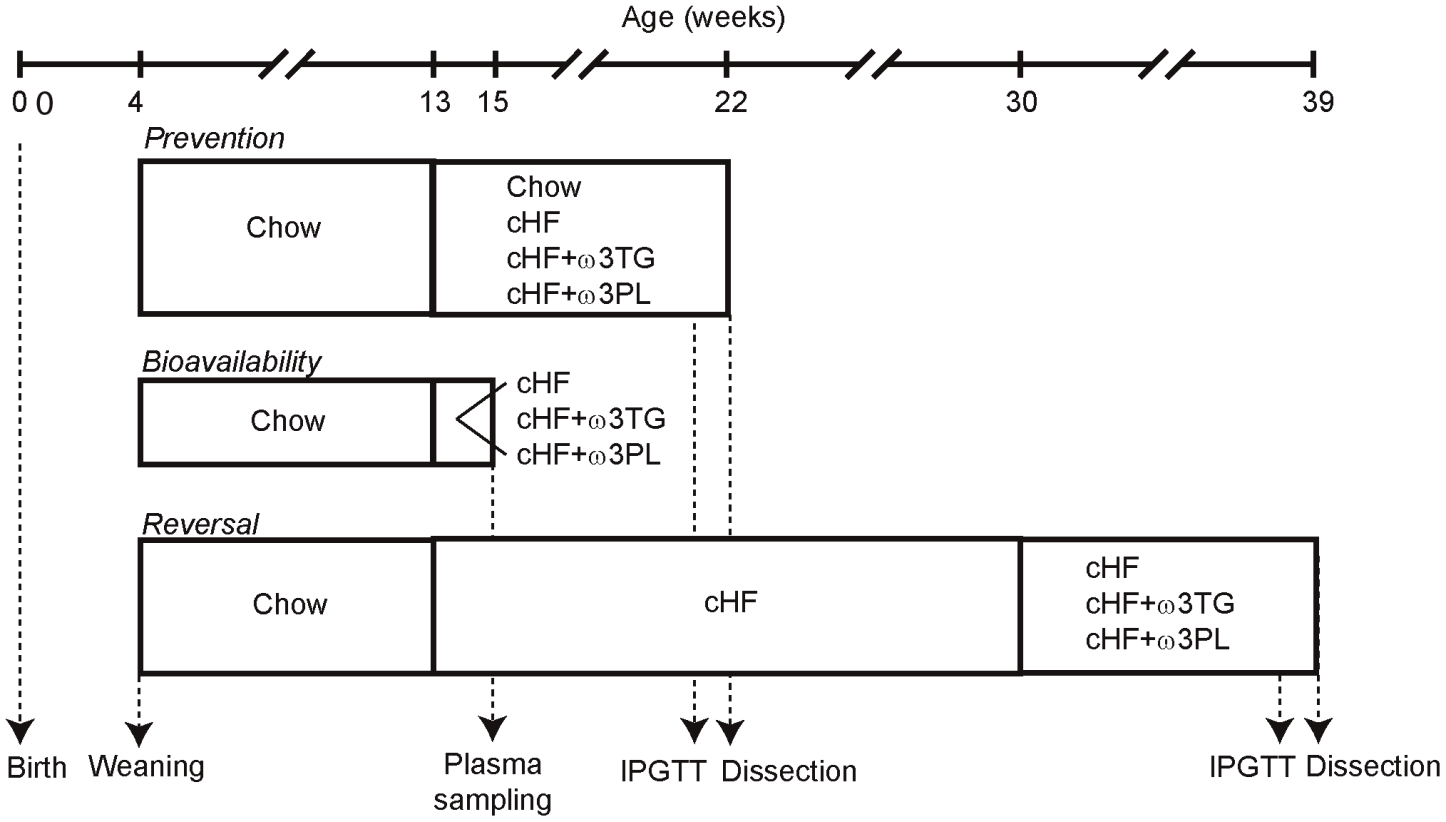
Timeline of dietary studies. All mice were weaned on Chow diet at 4 weeks of age. In the ‘prevention’ and ‘bioavailability’ studies, 3-month-old mice were switched from Chow to cHF, cHF+ω3TG and cHF+ω3PL diets for a period of 2 or 9 weeks, respectively, as indicated. In the ‘reversal study’, mice were fed cHF diet for a period of 4 month, followed by 9-week-feeding using cHF, cHF+ω3TG and cHF+ω3PL diets. In the ‘bioavailability’ study, cHF+ω3TG and cHF+ω3PL diets with several different concentrations of DHA and EPA were used (see Methods and Figure 2A,B), while in the ‘prevention’ and ‘reversal’ studies these diets were matched for their DHA/EPA content (i.e. 30 g DHA/EPA per kg diet), except for the ‘prevention’ study, in which a subgroup of mice was also treated using cHF+ω3PL diet containing 10 g DHA/EPA per kg diet. See Methods for further details. IPGTT, intraperitoneal (**i.p.**) glucose tolerance tests.

To characterise the efficacy of LC *n*-3 PUFA in the prevention of adverse consequences of high-fat intake (Fig. 1), three-month-old mice were fed for a 9-week-period either on a corn oil-based high-fat diet (**cHF** diet; lipids ∼ 35% wt/wt) or treated by isocaloric cHF-based diets with DHA/EPA supplemented as triglycerides (30 g DHA/EPA per kg diet; **cHF+ω3TG** diet) or phospholipids (10 or 30 g DHA/EPA per kg diet; **cHF+ω3PL** diet; Table S1, S2). Thus, in terms of fatty acid composition of experimental diets (Table S3), the ratio of total *n*-6 PUFA to total *n*-3 PUFA was 3.6 ∶ 1 and 2.9 ∶ 1 in the cHF+ω3TG and cHF+ω3PL diet (matched for DHA/EPA content of 30 g of DHA/EPA per kg diet), respectively. In contrast, this ratio was much higher (10.3 ∶ 1) in the cHF+ω3PL diet containing only 10 g of DHA/EPA per kg diet (Table S3).

No significant effects of dietary LC *n*-3 PUFA treatments on daily food intake were observed (Table 1). Compared with mice fed the low-fat standard diet (**Chow**; Table 1 and refs. [8]–[10], [12]), cHF feeding induced a moderate weight gain without a significant effect on blood lipid levels, but it altered glucose homeostasis. While the cHF+ω3TG-treatment mainly decreased plasma non-esterified fatty acid (**NEFA**) levels, the cHF+ω3PL-treatment showed a strong tendency to lower body weight gain and to reduce adiposity and adipocyte size, as well as exerting significant hypolipidemic effects (Table 1). Neither glycemia nor insulinemia were significantly changed in response to any of the treatments (Table 1). Importantly, glucose tolerance assessed as area under the glucose curve (**AUC**) was only improved in response to the cHF+ω3PL-treatment (Table 1 and Fig. S1), independently of the dietary DHA/EPA content. Both treatments tended to increase plasma levels of high-molecular weight (**HMW**) form of adiponectin. However, only the cHF+ω3PL-treatment resulted in lower lipid accumulation in the liver at both dietary DHA/EPA concentrations (Table 1).

**Table 1:** Prevention of obese phenotype by dietary LC *n*-3 PUFA administered as either triglycerides or phospholipids.

Diet	Chow	cHF	cHF+ω3TG	cHF+ω3PL	
DHA/EPA (g per kg diet)	-	-	30	10	30
*Energy balance*					
Body weight – initial (g)	29.4±0.7	28.2±1.2	27.5±0.8	27.2±0.9	28.0±0.6
Body weight – final (g)	31.2±0.7^d^	35.7±2.4	33.6±1.3	32.3±1.0	31.5±2.0
Weight gain (g)	1.82±0.67^d^	7.49±2.01	6.08±1.26	5.16±1.04	3.50±1.54
Food intake (kJ/day per animal)	ND	68±3	66±3	63±2	62±2
*WAT*					
Epididymal fat (mg)	303±26^d^	1268±349	1128±218	829±81	907±276
Adipocyte area (µm^2^)	ND	3413±544	2854±138	2398±168	2222±339
Subcutaneous fat (mg)	189±10^d^	414±81	402±59	331±18	356±67
*Lipid metabolites in plasma*					
Triacylglycerol (mmol/l)	0.96±0.05	1.12±0.13	0.91±0.13	1.13±0.11	0.71±0.13^a^
NEFA (mmol/l)	0.58±0.05	0.44±0.04	0.30±0.02^a^	0.47±0.05^b^	0.27±0.04^ac^
Cholesterol (mmol/l)	1.67±0.03^d^	3.34±0.34	2.92±0.28	3.04±0.22	2.94±0.30
*Glucose homeostasis*					
Glucose (mmol/l)	9.08±0.71	10.77±0.54	10.87±0.55	11.36±0.58	10.55±0.48
FBG (mmol/l)	4.75±0.39	5.15±0.22	5.34±0.26	4.82±0.20	4.70±0.17
AUC glucose (mmol · 180 min)	1517±92^d^	2221±78	2299±84	1760±63^ab^	1831±84^ab^
Plasma insulin (pmol/l)	97±9^d^	318±63	246±47	166±25	159±24
Adiponectin - HMW (A.U.)	ND	0.60±0.11	0.88±0.10	0.84±0.09	0.93±0.09
HMW: total	ND	0.37±0.04	0.44±0.04	0.44±0.03	0.47±0.03
*Tissue triacylglycerol*					
Liver (mg/g)	ND	41±7	36±4	22±2^a^	27±4^a^
Skeletal muscle (mg/g)	ND	23±3	17±4	11±2	17±3

Mice (3-months-old) were placed on various diets and killed after nine weeks of the dietary treatment. Body weight gain was calculated as the difference in body weight between the beginning of the experiment and after eight weeks of treatment. Food intake was monitored weekly during weeks 2 to 8 of the dietary treatment. As shown before [8], [58], there was no difference in food intake between the Chow- and cHF-fed mice. Glucose homeostasis was assessed by glucose tolerance test in mice fasted overnight after eight weeks of treatment (see Fig. S1). Data are means±SEM (*n* = 7).

a,b,c Significant differences (ANOVA) compared with cHF, cHF+ω3TG, and cHF+ω3PL (10 g per kg diet), respectively;

d *p*≤0.05 vs. cHF (t-test).

AU, arbitrary units; AUC, area under the glucose curve; FBG, fasting blood glucose; HMW: total, ratio of high molecular weight to total adiponectin; ND, not determined; NEFA, non-esterified fatty acids; WAT, white adipose tissue.

The above data documented that LC *n*-3 PUFA supplemented as phospholipids could preserve ‘healthy’ phenotype during the development of moderate obesity with greater efficacy than the triglyceride form.

### Bioavailability of DHA and EPA and Changes in Fatty Acid Composition of Tissue Lipids

**Figure 2:**
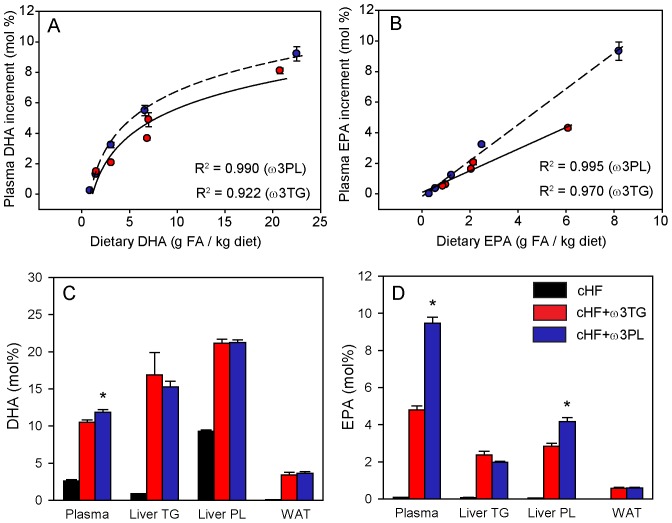
Bioavailability of DHA and EPA and their accumulation in the liver and WAT in the ‘prevention study’. **A**,**B** Fatty acid (**FA**) composition of total lipids in plasma was analyzed in mice fed for 2 weeks with the cHF+ω3TG (red circles) or cHF+ω3PL (blue circles) diet with various concentrations of DHA/EPA. The correlation plots between dietary content and the corresponding increments in plasma concentrations of DHA (**A**) and EPA (**B**) corrected for the values in the cHF-fed mice (DHA, 2.49–2.84 mol %; EPA, 0.05–0.16 mol %; see also Fig. 2C,D). The coefficient of determination (R^2^), describing the proportion of the dependent variable that is explained by the independent variable, is also shown. The concentration of DHA (**C**) and EPA (**D**) in total plasma lipids, liver triglycerides (**TG**) and phospholipid (**PL**) fraction, and in total lipids from epididymal white adipose tissue (**WAT**) was assessed in mice from the ‘prevention study’ following 9 weeks of feeding cHF+ω3TG (red bars) and cHF+ω3PL (blue bars) diets supplemented with 30 g DHA/EPA per kg, or the control cHF diet (black bars). For the detailed data on fatty acid composition, see Tables S4, S5, S6, S7). Data are means±SEM (*n* = 4−7); if SEM is not shown, it is smaller than the symbol size (**A,B**). *Significantly different from cHF+ω3TG (ANOVA).

In order to analyse the bioavailability of dietary DHA and EPA, the fatty acid composition of plasma lipids was evaluated by gas chromatography [36] in mice treated for two weeks with cHF+ω3TG and cHF+ω3PL diets at different levels of supplementation with the respective LC *n*-3 PUFA concentrate (Fig. 2). For both types of treatments, a positive correlation was found between dietary concentration and the increment in plasma DHA and EPA concentration, which was non-linear for DHA (Fig. 2A) and linear for EPA (Fig. 2B). Accordingly, in the prevention study, plasma concentrations of both DHA and EPA were higher in the cHF+ω3PL-treated mice than in the cHF+ω3TG-treated mice when compared at the same DHA/EPA dietary concentration (i.e. 30 g per kg diet). However, the difference was greater in the case of EPA (Fig. 2C,D and Table S4). At the tissue level, DHA as well as EPA concentrations in the triglyceride fraction either from the liver or total WAT lipids did not differ between the treatments; however, EPA was enriched in hepatic phospholipids of the cHF+ω3PL-treated mice (Fig. 2C,D and Tables S5, S6, S7).

A complex evaluation of fatty acid profiles in various lipid fractions carried out using orthogonal partial least squares-discriminant analysis (**oPLS-DA**; Fig. S2 A-D) revealed that, in the case of hepatic phospholipids, namely AA, DHA and EPA contributed to the separation between the diets (Fig. S2 A,B and Table S6). In the case of total WAT lipids, linoleic acid (**LA**; C18∶2), palmitic acid (C16∶0), oleic acid (C18∶1) and DHA contributed to the separation of all three dietary groups (Fig. S2 C,D), but only linoleic and palmitic acid levels differed significantly between the cHF+ω3TG- and cHF+ω3PL-treated mice (Table S7). This probably reflects the dietary content (Table S3).

**Figure 3:**
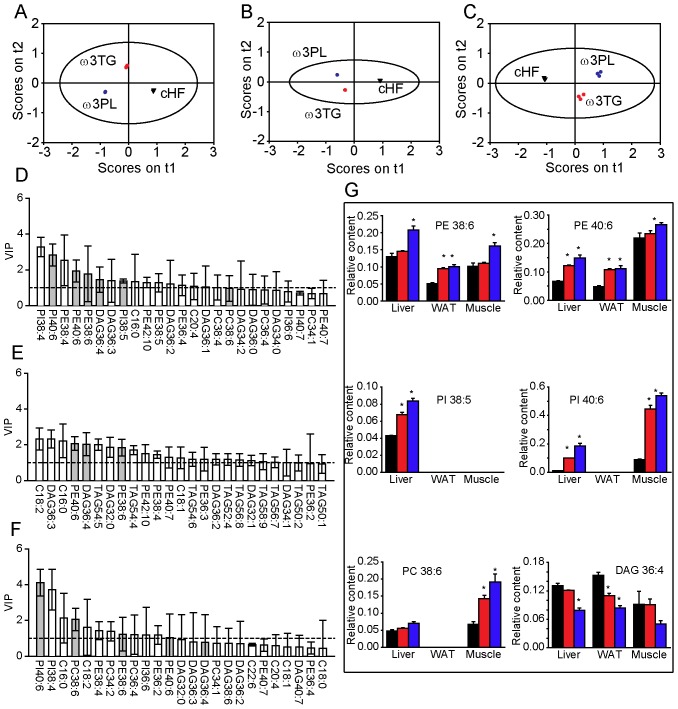
Changes in the lipid spectra in response to dietary LC *n*-3 PUFA in selected tissues of mice in the ‘prevention study’. Mice were fed for 9 weeks the cHF (black triangles), cHF+ω3TG (red circles), or cHF+ω3PL (blue circles) diet, while the last two diets were supplemented with 30 g DHA/EPA per kg. In total, 59, 71 and 61 lipid species were quantified in the liver, epididymal WAT and skeletal muscle, respectively, using time-of-flight secondary ion mass spectrometry (**TOF-SIMS**) analysis (see also Table S8, S9, S10). Means±SEM of 3–6 TOF-SIMS spectra from 3 animals per each group were used for orthogonal partial least squares-discriminant analysis (**oPLS-DA**). Score plots of the liver (**A**), WAT (**B**) and skeletal muscle (**C**) and corresponding variable important to projection (VIP) plots for the first latent variable (**D, E, F**), respectively. Only variables with VIP scores greater than 1 (denoted by a dashed horizontal line; **D-F**) and narrow confidence intervals were used for further evaluations, and only those discriminating between cHF+ω3TG and cHF+ω3PL diets (gray bars, **D-F**) were plotted (G; cHF diet, black bars; cHF+ω3TG diet, red bars; cHF+ω3PL diet; blue bars). See also Fig. S3 for additional lipid species identified. DAG, diacylglycerol. *Significantly different from cHF (all analyses by ANOVA).

The above results prompted us to increase the resolution of the analysis by performing time-of-flight secondary ion mass spectrometry (**TOF-SIMS**; [37], [38]) on the liver, WAT and skeletal muscle of animals fed on cHF+ω3TG and cHF+ω3PL diets (matched for the DHA/EPA content; 30 g per kg diet). This approach enabled the semi-quantitative detection of free fatty acids and phospholipid, triglycerides, and diacylglycerol species that differed in the total length of their side chains (for all the data, see Tables S8, S9, S10). Score plots showed a clear separation between the diets in all tissues examined (Fig. 3A–C). Next, lipid molecules were identified that distinguish the effects of diets within different tissues (Fig. 3D–F), with six of them showing a difference in concentration between the cHF+ω3TG- and cHF+ω3PL-treated mice in at least two of the tissues analysed (Fig. 3G). Several phospholipid species with polyunsaturated side chains probably containing DHA [i.e. phosphatidylethanolamine (**PE**) 38∶6, PE 40∶6, phosphatidylinositol (**PI**) 40∶6, and phosphatidylcholine (**PC**) 38∶6] or EPA (any of the above-mentioned analytes plus PI38∶5) as one of fatty acids bound, as well as diacylglycerol 36∶4 (possibly containing AA), discriminated between the cHF+ω3TG- and cHF+ω3PL-treated mice. For other less important lipid species that discriminated between the treatments, see Fig. S3.

Thus, the superior efficacy of dietary LC *n*-3 PUFA administered as phospholipids in terms of counteracting adverse effects of developing obesity was linked to the improved bioavailability of DHA and EPA, and to the accumulation of these fatty acids in phospholipids in metabolically relevant tissues. In a further experiment, we sought to characterise the effects of LC *n*-3 PUFA supplementation in a setting of established dietary obesity.

### Reversal of Dietary Obesity and Associated Disorders

Whether the lipid form of dietary LC *n*-3 PUFA could influence the efficacy of these fatty acids in reversing obesity and associated disorders was studied in mice fed a cHF diet for four months prior to dietary treatment by a cHF+ω3TG or cHF+ω3PL diet for nine weeks. In this experiment, cHF+ω3TG or cHF+ω3PL diets were matched for DHA/EPA content (30 g per kg). To mimic a typical situation in overweight or obese, insulin resistant type 2 diabetic subjects, all diets were also supplemented by metformin (2 g per kg). Of note, there were no significant differences between the control metformin-supplemented cHF diet and the cHF diet alone with regard to metabolic parameters shown in Table 2. Both cHF+ω3TG- and cHF+ω3PL-treatment decreased weight gain, with a stronger effect being exerted by the cHF+ω3PL-treatment, while food intake was not significantly affected by either treatment (Table 2). Both treatments reduced adiposity to a similar extent as well as the levels of plasma lipids, they suppressed glycemia in both fed and fasted mice, and they tended to improve glucose tolerance. Plasma insulin levels discriminated between the two treatments, with the cHF+ω3PL mice showing lower levels (Table 2). Both treatments induced adiponectin to a similar extent and lowered muscle lipid content (Table 2).

**Table 2:** Reversal of obese phenotype by dietary LC *n*-3 PUFA administered as either triglycerides or phospholipids.

Diet	cHF	cHF+ω3TG	cHF+ω3PL
*Energy balance*			
Body weight – initial (g)	40.2±1.5	38.5±1.9	41.2±1.1
Body weight – final (g)	46.0±2.2	41.1±2.3	41.2±1.5
Weight gain (g)	5.84±0.96	2.67±0.53^a^	0.04±0.65^ab^
Food intake (kJ/day per animal)	72±5	67±3	62±1
*WAT*			
Epididymal fat (mg)	2440±206	1906±231^a^	1978±164^a^
Subcutaneous fat (mg)	1288±188	1007±149	932±108
*Lipid metabolites in plasma*			
Triglycerides (mmol/l)	1.09±0.08	0.78±0.12^a^	0.66±0.04^a^
NEFA (mmol/l)	1.35±0.06	0.98±0.10^a^	0.94±0.04^a^
Cholesterol (mmol/l)	4.06±0.29	3.16±0.38^a^	3.37±0.20^a^
*Glucose homeostasis*			
Glucose (mmol/l)	11.92±0.70	9.74±0.37^a^	9.93±0.53^a^
FBG (mmol/l)	8.26±0.67	6.02±0.30^a^	5.94±0.33^a^
AUC glucose (mmol · 180 min)	3064±189	2650±179	2657±160
Plasma insulin (pmol/l)	307±55	223±52	156±18^a^
Adiponectin - HMW (A.U.)	0.54±0.05	1.01±0.12^a^	1.00±0.17^a^
HMW: total	0.42±0.02	0.51±0.02^a^	0.53±0.02^a^
*Tissue triglycerides*			
Skeletal muscle (mg/g)	29±6	13±2^a^	10±1^a^

To induce obesity, dyslipidemia and glucose intolerance, mice were fed a cHF diet between three and seven months of age, and then for nine more weeks on different diets (all containing 2 g metformin per kg diet). cHF+ω3TG and cHF+ω3PL diets were supplemented with ∼30 g DHA/EPA per kg diet. No significant differences between metformin-supplemented cHF diet and the cHF diet alone were observed with regard to any of the parameters shown above (e.g. cHF alone - weight gain: 46.0±2.0 g; epididymal WAT: 2160±204 mg; plasma triglycerides: 1.11±0.05 mmol/l; AUC glucose: 3395±231 mmol x 180 min; and plasma insulin: 400±47 pmol/l; not shown). Body weight gain was calculated as the difference in body weight between the beginning of the experiment and after eight weeks of treatment. Food intake was monitored weekly during weeks 2 to 8 of the dietary treatment. Glucose homeostasis was assessed by a glucose tolerance test in mice fasted overnight after eight weeks of treatment.

Data are means±SEM (*n* = 8–9).

a,b Significant differences (ANOVA) compared with cHF and cHF+ω3TG, respectively. AU, arbitrary units; AUC, area under the glucose curve; FBG, fasting blood glucose; HMW: total, ratio of high molecular weight to total adiponectin; NEFA, non-esterified fatty acids; WAT, white adipose tissue.

**Figure 4:**
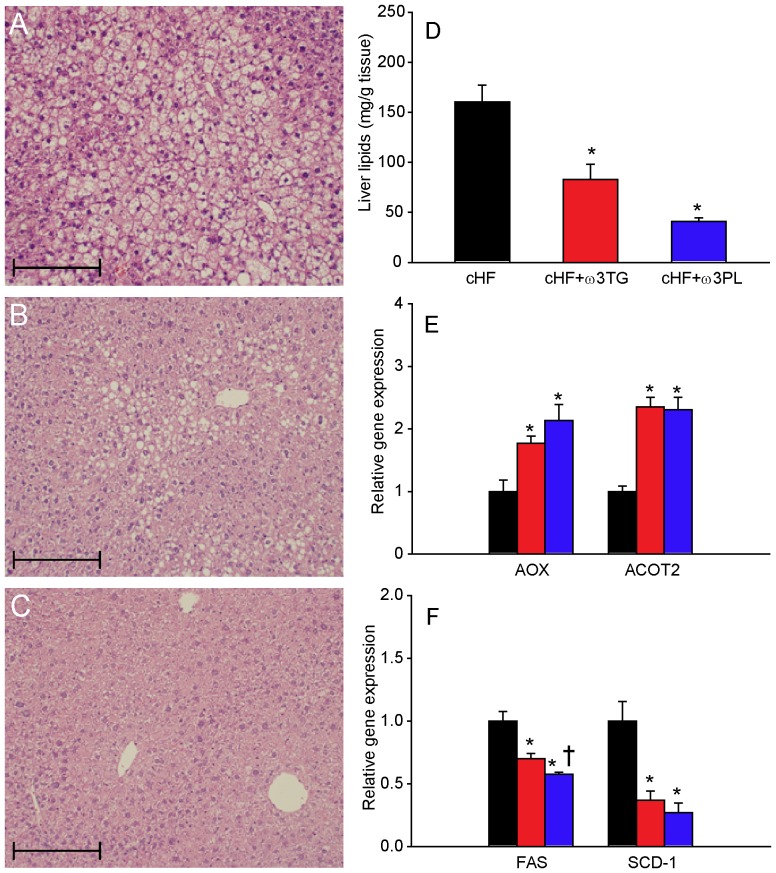
Reversal of hepatic lipid accumulation by dietary LC *n*-3 PUFA. Following a 4-month period of cHF-feeding, mice were fed for additional 9 weeks with cHF diet or treated using either cHF+ω3TG or cHF+ω3PL diet supplemented with 30 g DHA/EPA per kg; all diets also contained 2 g metformin per kg. Hematoxyline-eosin staining of liver sections from mice fed cHF (**A**), cHF+ω3TG (**B**), or cHF+ω3PL (**C**) diet. Hepatic lipid content (**D**). Quantification of mRNA levels of fatty acid oxidation (**E**) and lipogenic (**F**) genes in mice fed cHF (black bars), cHF+ω3TG (red bars), or cHF+ω3PL (blue bars) diets. **A-C**, Scale bars = 200 µm. AOX, acyl-CoA oxidase; ACOT2, acyl-CoA thioesterase 2; FAS; fatty acid synthase; SCD-1, stearoyl-CoA desaturase 1. Data are means±SEM (*n* = 7). *Significantly different from cHF (t-test or ANOVA); ^†^significantly different from cHF+ω3TG (ANOVA).

Histological analysis (Fig. 4A–C) and quantification of tissue lipids (Fig. 4D) documented that a cHF diet caused a marked hepatic steatosis (Fig. 4A), which was ameliorated by the cHF+ω3TG-treatment (Fig. 4B,D) and even more strongly by the cHF+ω3PL-treatment (Fig. 4C,D). These anti-steatotic effects were associated with increased hepatic expression of fatty acid oxidation genes (Fig. 4E) and down-regulation of lipogenic genes (Fig. 4F), while the latter effect was stronger in the cHF+ω3PL-treated mice.

**Figure 5:**
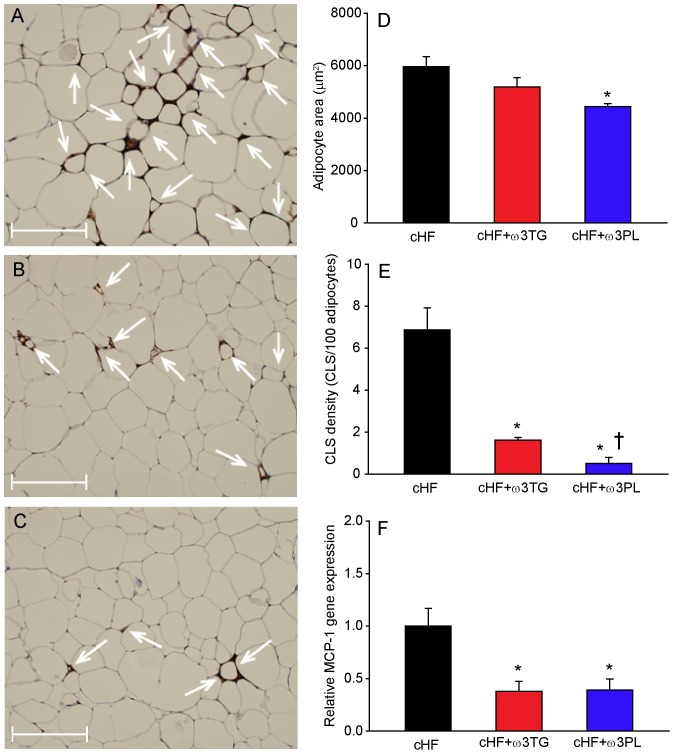
Reversal of adipose tissue hypertrophy and low-grade inflammation by dietary LC *n*-3 PUFA. Following a 4-month period of cHF-feeding, mice were fed for additional 9 weeks with cHF diet or treated using either cHF+ω3TG or cHF+ω3PL diet supplemented with 30 g DHA/EPA per kg; all diets also contained 2 g metformin per kg. Hematoxyline-eosin staining of epididymal fat sections from mice fed cHF (**A**), cHF+ω3TG (**B**), or cHF+ω3PL (**C**) diet. **D** Adipocyte size; morphometric analysis was based on more than 1,000 cells taken randomly from 5 different areas per animal (*n* = 3). **E** Amount of MAC-2 immunoreactive macrophages in the above tissue sections, expressed as the number of crown-like structures (**CLS**; indicated by white arrows in **A–C**). **F** Adipose tissue gene expression of monocyte chemoattractant protein-1 (**MCP-1**), a chemokine which is involved in the recruitment of monocytes to sites of injury and infection. cHF diet, black bars; cHF+ω3TG diet, red bars; cHF+ω3PL diet; blue bars. **A-C**, Scale bars = 200 µm. Data are means±SEM (n = 7−8; **D–F**). *Significantly different from cHF; ^†^significantly different from cHF+ω3TG (all analyses by ANOVA).

Immunohistochemical analysis of WAT (Fig. 5A–C) and adipocyte morphometry (Fig. 5D) revealed adipocyte hypertrophy in the cHF-fed mice (compare with Table 1), which was only significantly reduced by the cHF+ω3PL-treatment. WAT macrophage infiltration was significantly reduced by cHF+ω3TG-treatment and even more by cHF+ω3PL-treatment (Fig. 5E). Adipose tissue gene expression of monocyte chemoattractant protein-1 (**MCP1**; Fig. 5F) and macrophage marker CD68 (not shown) were reduced to a similar extent by both treatments.

The above data document similar beneficial effects of cHF+ω3TG- and cHF+ω3PL-treatment on obesity, lipid metabolism and glucose homeostasis in a reversal of obesity setting. However, the cHF+ω3PL-treatment was more efficient as regards the anti-steatotic effect on the liver and suppression of adipocyte hypertrophy and low-grade inflammation of WAT in obese mice.

### Endocannabinoids in Dietary Obese Mice

**Figure 6:**
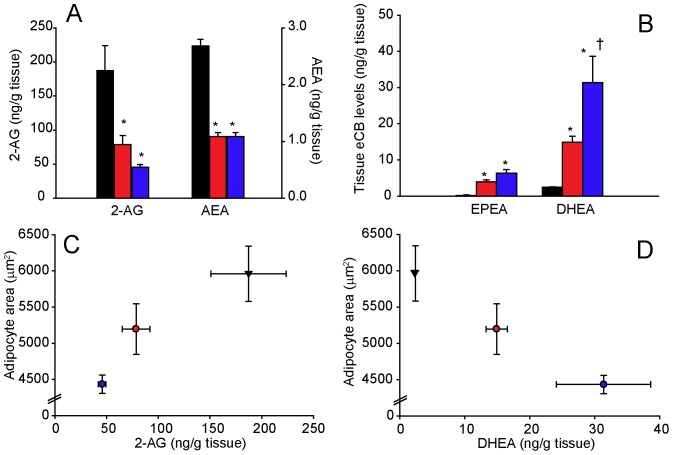
Modulation of adipose tissue levels of endocannabinoids and endocannabinoid-like molecules by dietary LC *n*-3 PUFA in the ‘reversal study’. Following a 4-month-period of cHF-feeding, mice were fed for additional 9 weeks cHF diet, or treated using either cHF+ω3TG or cHF+ω3PL diet supplemented with 30 g DHA/EPA per kg; all diets also contained 2 g metformin per kg. The effects of LC *n*-3 PUFA supplementation on adipose tissue levels of 2-arachidonoylglycerol (**2-AG**) and anandamide (**AEA; A**), as well as on the levels of *N*-eicosapentaenoylethanolamine (**EPEA**) and *N*-docosahexaenoylethanolamine (**DHEA; B**); cHF, black bars; cHF+ω3TG, red bars; cHF+ω3PL, blue bars. The relationship between adipocyte size and adipose tissue levels of either 2-AG (**C**) or DHEA (**D**) in mice fed cHF (black triangles), cHF+ω3TG (red circles), or cHF+ω3PL (blue circles) diet. Data are means±SEM (*n* = 8–9). *Significantly different from cHF (t-test or ANOVA); ^†^significantly different from cHF+ω3TG (ANOVA).

Reflecting (i) the pronounced differential effects of cHF+ω3TG- and cHF+ω3PL-treatment on the liver and WAT of mice in the reversal study, and (ii) the association of the beneficial effects of LC *n*-3 PUFA administered as phospholipids with the modulation of endocannabinoid system in WAT but not in the liver [26], the levels of endocannabinoids and endocannabinoid-like molecules were assessed in epididymal WAT of dietary obese mice (Fig. 6 and Table S11).

Compared with cHF-fed mice, only the cHF+ω3PL-treatment significantly reduced 2-AG levels (Fig. 6A). Levels of AEA, which were already relatively low in the cHF-fed mice, were further suppressed by both the cHF+ω3TG- and cHF+ω3PL-treatment. In contrast, tissue levels of *N*-eicosapentaenoylethanolamine (**EPEA**) and *N*-docosahexaenoylethanolamine (**DHEA**), i.e. anti-inflammatory molecules derived from EPA and DHA respectively, were induced by both treatments, with the cHF+ω3PL-treatment exerting a much stronger effect on DHEA (Fig. 6B). With the exception of dihomo-γ-linolenoylethanolamine, the WAT levels of other *N*-acyl ethanolamines did not change in response to LC *n*-3 PUFA supplementation (Table S11). The changes in the levels of endocannabinoids and related molecules in WAT closely reflected those observed in plasma (Table S11). Moreover, when the average values of 2-AG or DHEA levels in WAT were plotted against the size of adipocytes in different experimental groups, a positive relationship was observed in the case of 2-AG (Fig. 6C), while DHEA showed a negative relationship (Fig. 6D).

These data suggest that 2-AG rather than AEA could contribute to metabolic abnormalities in obese mice and that, compared with triglycerides, dietary LC *n*-3 PUFA administered as phospholipids are more effective in reducing 2-AG and increasing anti-inflammatory DHEA in WAT.

## Discussion

Given the well-known beneficial effects of LC *n*-3 PUFA on health (see the Introduction), it is of the utmost importance to define the optimal lipid form for delivering these fatty acids into the organism. We show here that dietary DHA and EPA supplemented as phospholipids were more efficient than triglycerides in ameliorating obesity-associated pathologies, including impaired glucose homeostasis, hepatic steatosis and dyslipidemia, as well as adipocyte hypertrophy and low-grade inflammation of WAT in mice fed a high-fat diet.

It is relatively difficult to compare the biological effectiveness of individual components of complex mixtures, as in case of the LC *n*-3 PUFA concentrates from marine fish used here. Thus, in the ‘prevention study’, assuming that LC *n*-3 PUFA are the major active constituents, cHF+ω3TG and cHF+ω3PL treatments were compared using diets matched for total DHA/EPA content (30 g per kg diet). Due to a higher DHA/EPA level in the triglyceride than in the phospholipid concentrate, a higher portion of dietary lipids had to be replaced by the phospholipid concentrate in order to achieve an equal dietary DHA/EPA content. Despite this fact, the ratios of total *n*-6 PUFA to *n*-3 PUFA in the two diets were quite similar. In order to eliminate a possible confounding effect of differential dilutions of corn oil (high in LA, i.e. *n*-6 PUFA) as in the case of triglyceride and phospholipid concentrates, the cHF+ω3PL-treatment was also performed at a 3-fold lower dietary DHA/EPA content (10 g DHA/EPA per kg diet), i.e. ∼30% less dietary lipids were replaced by the phospholipid concentrate as compared with the cHF+ω3TG diet containing 30 g DHA/EPA per kg diet. Irrespectively of the DHA/EPA content, both cHF+ω3PL treatments preserved glucose tolerance and prevented hepatic steatosis, while the cHF+ω3TG-treatment did not. This made it possible to exclude the possibility that a higher efficacy of the phospholipid-based treatment was the result of a higher content of the concentrate in the diet rather than of qualitative differences between the LC *n*-3 PUFA concentrates. Moreover, when using the DHA/EPA-matched diets (i.e. 30 g DHA/EPA per kg), the cHF+ω3PL-treatment tended to exert stronger beneficial effects on weight gain, adiposity and fat cell size, plasma triglycerides, fasting glycemia and insulinemia.

In the ‘reversal study’, body weight, hepatic steatosis, adipocyte hypertrophy and low-grade inflammation of WAT were more potently reduced by the cHF+ω3PL-treatment. In contrast to the prevention study, blood glucose levels in obese mice were lowered to a similar extent by both treatments. However, only the cHF+ω3PL-treatment significantly decreased plasma insulin. Future experiments should therefore focus on detailed characterisation of changes in insulin sensitivity brought about by dietary phospholipids.

In line with previous studies [30]–[34], our results document better bioavailability of both DHA and EPA when supplemented as dietary phospholipids and this effect can largely be attributed to a marked improvement in EPA bioavailability (see also ref. [34]). Accordingly, in humans, most orally administered PC is hydrolysed to lysoPC before absorption [39]. This in turn is well absorbed and may augment LC *n*-3 PUFA delivery [40]. However, similar ratio between EPA and DHA content was found in the PC and the lysoPC fraction from the marine phospholipid concentrate used in our study, suggesting that the differences in EPA and DHA bioavailability could not be explained by their absorption via the lysoPC molecule.

In agreement with the previous observation in both humans [41] and mice [42], saturable incorporation of DHA into total plasma lipids was observed, while a linear relationship between dietary concentration of EPA and its plasma level was found, which contributed to a relatively high bioavailability of EPA from the phospholipid concentrate. The difference in bioavailability was not reflected in changes in DHA and EPA in the composition of either liver or WAT triglycerides. However, in total liver phospholipids, EPA accumulated more and AA less in the cHF+ω3PL-treated mice than in the cHF+ω3TG-treated mice. The differences between the two treatments became apparent when the TOF-SIMS analysis was conducted. PE, PI and PC phospholipid species, such as PE 38∶6, PE 40∶6 and PI 38∶5 in the liver and PE 40∶6 in WAT, were the discriminating analytes. It is likely that these phospholipid species are enriched with DHA, since it has previously been shown that, in egg phospholipids from hens fed on seal blubber oil [43], [44], there is a preferential incorporation of DHA into the PE, PI, and PC fraction [43], especially in the sn-2 position of the phospholipid molecule [44]. These data are consistent with the notion that cell membrane phospholipids represent an important reservoir of DHA and EPA and their processing into lipid signalling molecules [9], [20], [22], and they suggest that the difference in the effectiveness of the treatments is largely explained by differential accumulation of DHA and EPA in specific phospholipid molecules (see also [4]). It is also likely that the more potent reductions of AA in the phospholipid and diacylglycerol pools associated with (i) AA replacement in membrane phospholipids by LC *n*-3 PUFA (for reviews, see [45], [46]), and (ii) lower dietary content of LA, which is the precursor of AA, contribute to the superior anti-inflammatory effects of the cHF+ω3PL-treatment. This could be due to a lower formation of both, the pro-inflammatory mediators and endocannabinoids derived from AA.

In line with previous data on krill oil [11], [25], [26], [35], our results support the notion that the endocannabinoid system is involved in the mechanism of action of LC *n*-3 PUFA. Levels of 2-AG in WAT were more efficiently reduced by LC *n*-3 PUFA administered as phospholipids, which was paralleled by decreased levels of AA in the diacylglycerol fraction, attesting to the latter’s precursor role in 2-AG synthesis [23]. In contrast to another report [25], the levels of AEA in WAT were relatively low and were further reduced by LC *n*-3 PUFA in the diet. Therefore, as is the case in humans [24], it is unlikely that AEA contributes significantly to metabolic derangements in dietary obese mice. Dietary phospholipids also induced adipose levels of *N*-acyl ethanolamines EPEA and especially DHEA, i.e. the metabolites of EPA and DHA, respectively, more strongly and effectively than triglycerides. The pathophysiological role of the endocannabinoid system in the development of adipose tissue inflammation [47] or hepatic steatosis [48], [49] is well described, and anti-inflammatory effects of adipocyte-derived EPEA or DHEA have recently been suggested [50], [51]. Average daily food intake was slightly but insignificantly decreased in the cHF+ω3PL-treated mice, however the levels of *N*-oleoylethanolamine, i.e. the endocannabinoid that is known to suppress food intake [52], were completely unchanged both in WAT and plasma of these mice. Thus, our results are in agreement with a specific role of the dysregulated endocannabinoid system in WAT in the pathophysiology of metabolic syndrome [24], [26].

In fact, the increase in obesity prevalence in the United States during the 20th century could be in part due to the elevated consumption of LA, the precursor of AA, and hence also the precursor of the AA-derived endocannabinoids [53], [54]. Very recently, this idea was supported by dietary experiments in mice fed various diets differing in the LA content [54]. This content was positively correlated with the levels of AA, 2-AG and AEA in phospholipids isolated both from the liver and erythrocytes, while dietary LA also promoted accumulation of body fat. In accordance with our results in plasma and WAT, addition of EPA and DHA to the diet resulted in a decrease of endocannabinoid levels in the liver and also in hypothalamus of mice fed experimental diets with a high LA content [54]. Accordingly, we have observed before in mice that supplementation of a high-fat diet by LC *n*-3 PUFA, under very similar conditions as used in the ‘prevention study’ here, reduced body fat depots when LA formed 53% of dietary lipids, but did not affect adiposity when the LA content represented 15% of dietary fat [55]. Taken together, the data further support the role of dietary LA as the key factor controlling the activity of the endocannabinoid system, and the attenuation of the endocannabinoid system activity as the key mechanism underlying anti-obesity effects of dietary LC *n*-3 PUFA supplementation under these conditions. However, precise involvement of various tissues in the metabolic impact of attenuated endocannabinoid system activity in response to LC *n*-3 PUFA should be better characterized [23]. Moreover, conflicting data exist regarding the potential role of dietary macronutrient composition in the anti-obesity effects of LC *n*-3 PUFA [56], [57]. Nevertheless, the data also suggest that the experimental model of obesity induced in mice by feeding a high-fat diet containing high levels of LA, as used in our current study as well as in most of our previous studies [8]–[10], [12], [58], is highly relevant for understanding the beneficial metabolic effects of LC *n*-3 PUFA supplementation in humans consuming an obesogenic Western diet.

Cannabinoid receptor 1 antagonist rimonabant has been used to effectively treat obesity. It had to be withdrawn from clinical practice, however, due to adverse side effects on the central nervous system. Selective blocking of the peripheral endocannabinoid system might nevertheless represent an effective and safe therapy [24], [59]. In this respect, modulation of the endocannabinoid system in the peripheral tissues by LC *n*-3 PUFA administered as phospholipids should be further explored. With regard to treatment strategies for obesity-associated disorders, combined treatment using LC *n*-3 PUFA as triglycerides and pharmaceuticals like thiazolidinediones has been shown to be very effective [8], [58]. The inclusion of LC *n*-3 PUFA as phospholipids might increase the efficacy of such treatment by more potently modulating the activity of the peripheral endocannabinoid system.

To preserve health, most of the nutritional and cardiological societies worldwide recommend to increase intake of fatty sea fish, which contain LC *n*-3 PUFA in both the triacylglycerol and phospholipid form. Nutritional supplements containing LC *n*-3 PUFA as triglycerides (or ethyl-esters) are also considered [60]. Our results are in favour of this strategy and they suggest that the efficacy of the supplements could be further augmented based on the use of marine-derived phospholipids.

In conclusion, compared with triglycerides, dietary LC *n*-3 PUFA as phospholipids from marine fish exert superior metabolic effects in the context of high-fat diet-induced obesity in mice. Dietary supplementation of relatively low doses of DHA and EPA administered as phospholipids resulted in better bioavailability, while more efficacious lowering of the AA content in cellular phospholipids resulted in reduced production of the classical endocannabinoids AEA and 2-AG and increased production of anti-inflammatory molecules such as EPEA and DHEA. By multiple mechanisms of action, dietary phospholipids of marine origin might thus substantially improve prevention and treatment strategies for obesity-associated metabolic disorders.

## Materials and Methods

### Animals and Treatments

Male C57BL/6J mice (Institute of Physiology, Academy of Sciences of the Czech Republic, Prague) were weaned on a standard Chow (extruded Ssniff R/M-H diet; Ssniff Spezialdieten GmbH, Soest, Germany) and maintained on a 12∶12-hr light-dark cycle at 22°C (3–4 animals/cage). To induce obesity, three-month-old mice were assigned to cHF diet (lipids ∼35% wt/wt; mostly corn oil; virtually DHA/EPA-free; see also ref. [8]). Three different dietary treatment protocols were used (Fig. 1):

Firstly, a ‘prevention study’ was performed to characterise the effects of LC *n*-3 PUFA on the development of obese phenotype, while replacing part of corn oil in the cHF diet with the LC *n*-3 PUFA concentrates, either as triacylglycerols (DHA, 46% wt/wt; EPA, 14% wt/wt; product EPAX 1050 TG; EPAX AS, Aalesund, Norway; cHF+ω3TG diet) or phospholipids from marine fish (DHA, 17–20% wt/wt; EPA, 5–8% wt/wt; prepared by EPAX AS; see Tables S1 and S2; cHF+ω3PL diet) in order to achieve a sum of DHA and EPA (DHA/EPA) of 30 g per kg diet. A group of mice was also treated using a cHF+ω3PL diet containing 10 g DHA/EPA per kg diet. For the fatty acid composition of experimental diets, see Table S3. The study lasted for a period of 9 weeks and the cHF-fed mice served as controls. Chow-fed mice were also analysed. The energy density of the Chow and isocaloric cHF-based diets was 16.3 and 22.8 kJ per g diet, respectively.

Secondly, a ‘bioavailability study’ was performed that was similar to that described above, except that (i) various formulations of the cHF+ω3TG and cHF+ω3PL diet were used, in which the DHA and EPA content varied from 0.9 to 22.5 g DHA per kg diet and from 0.3 to 8.2 g EPA per kg diet; (ii) these dietary treatments lasted for only two weeks, and (iii) the cHF-fed but not Chow-fed mice were used, and served as controls.

Thirdly, in a ‘reversal study’, obesity was induced by cHF feeding between three and seven months of age prior to 9-week-long treatment using cHF+ω3TG or cHF+ω3PL diets supplemented with 30 g DHA/EPA per kg diet; the cHF-fed mice served as controls. In this study, all the diets (i.e. cHF, cHF+ω3TG, and cHF+ω3PL) also contained 2 g metformin per kg diet (Tables S1,S2).

Body weight was monitored weekly, while a fresh ration was given every two days. The food intake of a group of mice (3 to 4 mice/cage), assessed once weekly during a 24-hr period, was averaged per mouse during weeks 2 to 8 after the start of differential dietary treatment. Mice were killed by cervical dislocation under diethylether anesthesia between 9 and 11 a.m. Dorsolumbar (subcutaneous) and epididymal (abdominal) WAT, the liver and skeletal muscle (*m. quadriceps femoris*) were dissected and snap-frozen in liquid nitrogen. Tissues and EDTA-plasma from truncal blood were stored at –70°C. Animal experiments were approved by the Animal Care and Use Committee of the Institute of Physiology Academy of Sciences of the Czech Republic v.v.i. (Approval Number: 58/2009) and followed the guidelines.

### Plasma Metabolites, Hormones and Enzymes

Plasma triglycerides, total cholesterol, NEFA and insulin were determined as before [8]. The distribution of adiponectin multimeric complexes in plasma was determined by Western blotting [61].

### Glucose Tolerance Test

An intraperitoneal glucose tolerance test was performed on fasted mice as described before [12].

### Quantitative RT-PCR-based Analysis

An analysis of levels of various transcripts in total RNA isolated from liver and epididymal fat was performed and normalised as before [3]. The sequences of oligonucleotide primers have been published before [8], [9].

### Tissue Lipid Content

Liver and muscle lipid content was estimated in ethanolic KOH tissue solubilisates [12].

### Light Microscopy and Immunohistochemical Analysis

Samples of epididymal fat and liver were fixed in 4% formaldehyde, embedded in paraffin, and 5 µm-sections were stained using hematoxylin-eosine. In epididymal fat, a macrophage marker, MAC-2/galectin-3, was also detected using specific antibodies [8]. Digital images were captured using Olympus AX70 light microscope and a DP 70 camera (Olympus, Tokyo, Japan). Adipocyte morphometry was performed using a Lucia IMAGE version 4.81 (Laboratory Imaging, Prague, Czech Republic).

### Plasma and Tissue Fatty Acid Composition in Lipid Fractions

The fatty acid composition of total lipid fraction from diets, plasma and epididymal fat, as well as of triglyceride and total phospholipid fractions from the liver, was analysed using gas chromatography [36]. TOF-SIMS analysis [37], [38] was applied to analyse free fatty acids and the fatty acid composition of the triglyceride, diacylglycerol, PC, PE and PI fractions from the liver, epididymal fat and skeletal muscle.

### Endocannabinoid Levels

The levels of 2-AG, AEA, DHEA, EPEA, oleoylethanolamine, palmitoylethanolamine, stearoylethanolamine and dihomo-γ-linolenoylethanolamine were quantified, either in individual samples of epididymal fat (∼100 mg) or in pooled samples (∼0.1 ml) of EDTA-plasma using LC-MS/MS. Tissues and plasma were extracted with acetonitrile containing the appropriate internal standards. Tissue extracts were analysed on a Quantum TSQ (Thermo, Breda, The Netherlands) LC-MS, while plasma extracts were analysed on a Xevo TQ-S (Waters, Etten-Leur, The Netherlands) LC-MS.

### Statistical Analysis

All values are presented as means±SEM. Logarithmic transformation was used to stabilise variance in cells when necessary. Data were analysed by paired t-test or ANOVA (one-way or two-way) with Holm-Sidak post-hoc tests using SigmaStat 3.5 statistical software. Comparisons were judged to be significant at *p*≤0.05. Lipidomic data were evaluated using oPLS-DA and Umetrics SIMCA-P+12 statistical software (Umetrics AB, Umeå, Sweden).

## Supporting Information

Figure S1 **Glucose tolerance** **in response to dietary LC** ***n*** **-3 PUFA administered to mice in the ‘prevention study’.** Mice were fed for 9 weeks a corn oil-based high-fat (cHF; black circles) diet, or experimental cHF+ω3TG (red circles) and cHF+ω3PL (blue triangles) diets containing 30 g DHA/EPA per kg diet. Some mice were also fed a low-fat Chow diet (grey circles). Plasma glucose profiles during 180 min following i.p. injection of glucose (time 0). Data are means±SEM.
(TIF)

Figure S2 **Orthogonal Partial Least Squares - Discriminant Analysis (Opls-DA) of fatty acid composition data in the liver and adipose tissue of mice in the ‘prevention study’.** Mice were fed for 9 weeks a corn oil-based high-fat (cHF; black triangles) diet or cHF-based experimental diets, in which part of dietary lipids was replaced by LC *n*-3 PUFA concentrates either in the form of triglycerides (cHF+ω3TG; red circles) or marine phospholipids (cHF+ω3PL; blue circles) to achieve dietary EPA and DHA concentration of 30 g per kg diet. In total, 22 and 17 fatty acids were quantified in the phospholipid fraction from the liver and in total lipids from adipose tissue, respectively, using gas chromatography (see also Tables S6 and S7). To identify the major fatty acids discriminating between the cHF+ω3TG and cHF+ω3PL groups, multivariate analysis was performed on fatty acid profiles in liver phospholipids (**A**,**B**) and total lipids in adipose tissue (**C**,**D**) using the oPLS-DA algorithm. Within each tissue, mice (*n* = 4−7) were separated into 3 distinct groups based on the diet (**A**,**C**), and variables important to the projection of the first latent variable were plotted (**B**,**D**). To identify the most important ones, only variables with VIP scores greater than 1 (denoted by a solid horizontal line; **B** and **D**) and narrow confidence intervals were used for further evaluations (interpretations).
(TIF)

Figure S3 **The levels of lipid species discriminating betweeen the phospholipid and triglyceride LC** ***n*** **−3 PUFA supplementation in the liver, adipose tissue and muscle of mice in the ‘prevention study’.** Mice were fed for 9 weeks a corn oil-based high-fat (cHF; black bars) diet or cHF-based experimental diets, in which part of dietary lipids was replaced by LC *n*−3 PUFA concentrates either in the form of triglycerides (cHF+ω3TG; red bars) or phospholipids (cHF+ω3PL; blue bars) to achieve dietary EPA and DHA concentration of 30 g per kg diet. In total, 59, 71 and 61 lipid species were quantified in the liver, adipose tissue and skeletal muscle, respectively, using the TOF-SIMS analysis (see also Tables S8−S10). Major lipid species discriminating between the cHF+ω3TG and cHF+ω3PL groups were identified by the oPLS-DA algorithm.
(TIF)

Table S1 **Diets and treatments.** cHF-based experimental diets were supplemented with the EPA and DHA concentrates either in the form of triglycerides (cHF+ω3TG diet) or marine phospholipids (cHF+ω3PL diet) to achieve various dietary EPA and DHA concentrations. In the ‘reversal study‘, all diets were also supplemented by 2 g metformin per kg diet. The concentrates were dissolved in corn oil prior to the preparation of experimental diets. ^a^Percentage of dietary lipids replaced by EPAX 1050 TG (containing 46% DHA and 14% EPA). ^b^Percentage of dietary lipids replaced by marine phospholipids (containing 20% DHA and 8% EPA). ^c^Percentage of dietary lipids replaced by EPAX 1050 TG (containing 46% DHA and 14% EPA). ^d^Percentage of dietary lipids replaced by marine phospholipids (containing 17% DHA and 5% EPA). See Table S2 for further details on the composition of marine phospholipids. -, not used in the respective study.
(DOC)

Table S2 **Phospholipid and fatty acid composition of the EPA and DHA concentrate based on marine phospholipids.** Food grade herring meal was extracted once with ethanol (ethanol/meal = 5∶1 vol/wt) at 60°C. The extract was concentrated to dryness in vacuo on a rotary evaporator. The resulting residue was treated with ice cold acetone to extract the bulk of neutral lipids from the marine phospholipid concentrate with the EPA and DHA content of 50−80 and 170−200 mg per g diet, respectively. Total phospholipid content in the marine phospholipid concentrate was 74−80% (of three different analyses), while the content of major phospholipid fractions in the concentrate was as follows: phosphatidylcholine (PC; 47−56%), lysoPC (∼3%), phosphatidylethanolamine (PE; 9−13), lysoPE (2−3%). Other minor phospholipid fractions in the product include phosphatidylinositol (∼1%), sphingosine (2−4%), and unidentified fraction (2–9%). Fatty acid composition (mol %) of a typical batch determined by gas chromatography is shown. MUFA, monounsaturated fatty acids. –, ≤0.1% (detection limit).
(DOC)

Table S3 **Fatty acid composition of experimental diets in the ‘prevention study’.** cHF-based experimental diets were supplemented with the EPA and DHA concentrates either in the form of triglycerides (cHF+ω3TG diet) or marine phospholipids (cHF+ω3PL diet) to achieve various dietary EPA and DHA concentrations. Fatty acid composition (mol %) was analyzed in triplicates in the total lipid fraction extracted from the experimental diets. Standard errors are not shown, but for most fatty acids represented <5% of the mean. SFA, saturated fatty acids; MUFA, monounsaturated fatty acids; PUFA, polyunsaturated fatty acids. –, ≤0.1% (detection limit).
(DOC)

Table S4 **Fatty acid composition of plasma in the ‘prevention study’.** Fatty acid composition was analyzed in the total lipid fraction extracted from plasma. The results (mol %) are expressed as means ± SEM (*n* = 4). ^a,b,c^Significant differences (ANOVA) compared with cHF, cHF+ω3TG, and cHF+ω3PL (10 g per kg diet), respectively. Similar results were obtained when fatty acid composition in plasma was analyzed already after 2 weeks of dietary intervention (not shown). MUFA, monounsaturated fatty acids; PUFA, polyunsaturated fatty acids. –, ≤0.1% (detection limit).
(DOC)

Table S5 **Fatty acid composition of total triglycerides in the liver from the ‘prevention study’.** Fatty acid composition was analyzed in the triglyceride fraction extracted from the liver. The results (mol %) are expressed as means ± SEM (n = 4). a,b,cSignificant differences (ANOVA) compared with cHF, cHF+ω3TG, and cHF+ω3PL (10 g per kg diet), respectively. SFA, saturated fatty acids; MUFA, monounsaturated fatty acids; PUFA, polyunsaturated fatty acids. –, ≤0.1% (detection limit).
(DOC)

Table S6 **Fatty acid composition of total phospholipids in the liver from the ‘prevention study’.** Fatty acid composition was analyzed in the total phospholipid fraction extracted from the liver. The results (mol %) are expressed as means ± SEM (*n* = 4). ^a,b,c^Significant differences (ANOVA) compared with cHF, cHF+ω3TG, and cHF+ω3PL (10 g per kg diet), respectively. SFA, saturated fatty acids; MUFA, monounsaturated fatty acids; PUFA, polyunsaturated fatty acids. –, ≤0.1% (detection limit).
(DOC)

Table S7 **Fatty acid composition in total lipids from adipose tissue in the ‘prevention study’.** Fatty acid composition was analyzed in the total lipid fraction extracted from abdominal adipose tissue (epididymal fat depot). Results (mol %) are expressed as means ± SEM (*n* = 7). ^a,b,c^Significant differences (ANOVA) compared with cHF, cHF+ω3TG, and cHF+ω3PL (10 g per kg diet), respectively. MUFA, monounsaturated fatty acids; PUFA, polyunsaturated fatty acids. –, ≤0.1% (detection limit).
(DOC)

Table S8 **TOF-SIMS analysis of lipid fractions in the liver from the ‘prevention study’.** Various lipid species were analyzed in the liver by the TOF-SIMS method. Data are expressed as the (cHF+ω3TG)/cHF and (cHF+ω3PL)/cHF normalised signal intensity ratios for lipid signals, originating from mice fed the control diet (cHF) and from mice fed the cHF-based experimental diets supplemented with the EPA and DHA concentrate either in the form of triglycerides (cHF+ω3TG) or marine phospholipids (cHF+ω3PL). DAG, diacylglycerol; PC, phosphatidylcholine; PE, phosphatidylethanolamine; PI, phosphatidylinositol.
(DOC)

Table S9 **TOF-SIMS analysis of lipid fractions in adipose tissue from the ‘prevention study’.** Various lipid species were analyzed in epididymal adipose tissue by the TOF-SIMS method. Data are expressed as the (cHF+ω3TG)/cHF and (cHF+ω3PL)/cHF normalised signal intensity ratios for lipid signals, originating from mice fed the control diet (cHF) and from mice fed the cHF-based experimental diets supplemented with the EPA and DHA concentrate either in the form of triglycerides (cHF+ω3TG) or marine phospholipids (cHF+ω3PL). DAG, diacylglycerol; PE, phosphatidylethanolamine.
(DOC)

Table S10 **TOF-SIMS analysis of lipid fractions in the skeletal muscle from the ‘prevention study’.** Various lipid species were analyzed in the skeletal muscle (*m. quadriceps femoris*) by the TOF-SIMS method. Data are expressed as the (cHF+ω3TG)/cHF and (cHF+ω3PL)/cHF normalised signal intensity ratios for lipid signals, originating from mice fed the control diet (cHF) and from mice fed the cHF-based experimental diets supplemented with the EPA and DHA concentrate either in the form of triglycerides (cHF+ω3TG) or marine phospholipids (cHF+ω3PL). DAG, diacylglycerol; PC, phosphatidylcholine; PE, phosphatidylethanolamine; PI, phosphatidylinositol.
(DOC)

Table S11 **The levels of endocannabinoids and endocannabinoid-like molecules in plasma and epididymal adipose tissue of dietary obese mice from the ‘reversal study’.** To induce obesity, dyslipidemia and glucose intolerance, mice were fed the cHF diet between 3 and 7 months of age, and then for 9 more weeks by different diets (all containing 2 g metformin per kg diet). cHF+ω3TG and cHF+ω3PL diets were supplemented with ∼30 g DHA/EPA per kg diet. Data (ng/g tissue) are expressed as means±SEM (plasma, *n* = 2 of pooled samples from 3 mice; adipose tissue, *n* = 8−9). ^a^Significant differences compared with cHF (t-test or ANOVA). ^b^Significant differences compared with cHF+ω3TG (ANOVA). 2-AG, 2-arachidonoylglycerol; AEA, anandamide; DHEA, N-docosahexaenoylethanolamine; EPEA, N-eicosapentaenoylethanolamine; OEA, oleoylethanolamine; PEA, palmitoylethanolamine; SEA, stearoylethanolamine; DLE, dihomo-γ-linolenoylethanolamine.
(DOC)
