# Supplementary figures and images for: Metabolic Effects of n-3 PUFA as Phospholipids Are Superior to Triglycerides in Mice Fed a High-Fat Diet: Possible Role of Endocannabinoids
Source: PLoS One. 2012 Jun 11;7(6):e38834. doi: 10.1371/journal.pone.0038834 (PMC3372498; doi:10.1371/journal.pone.0038834)

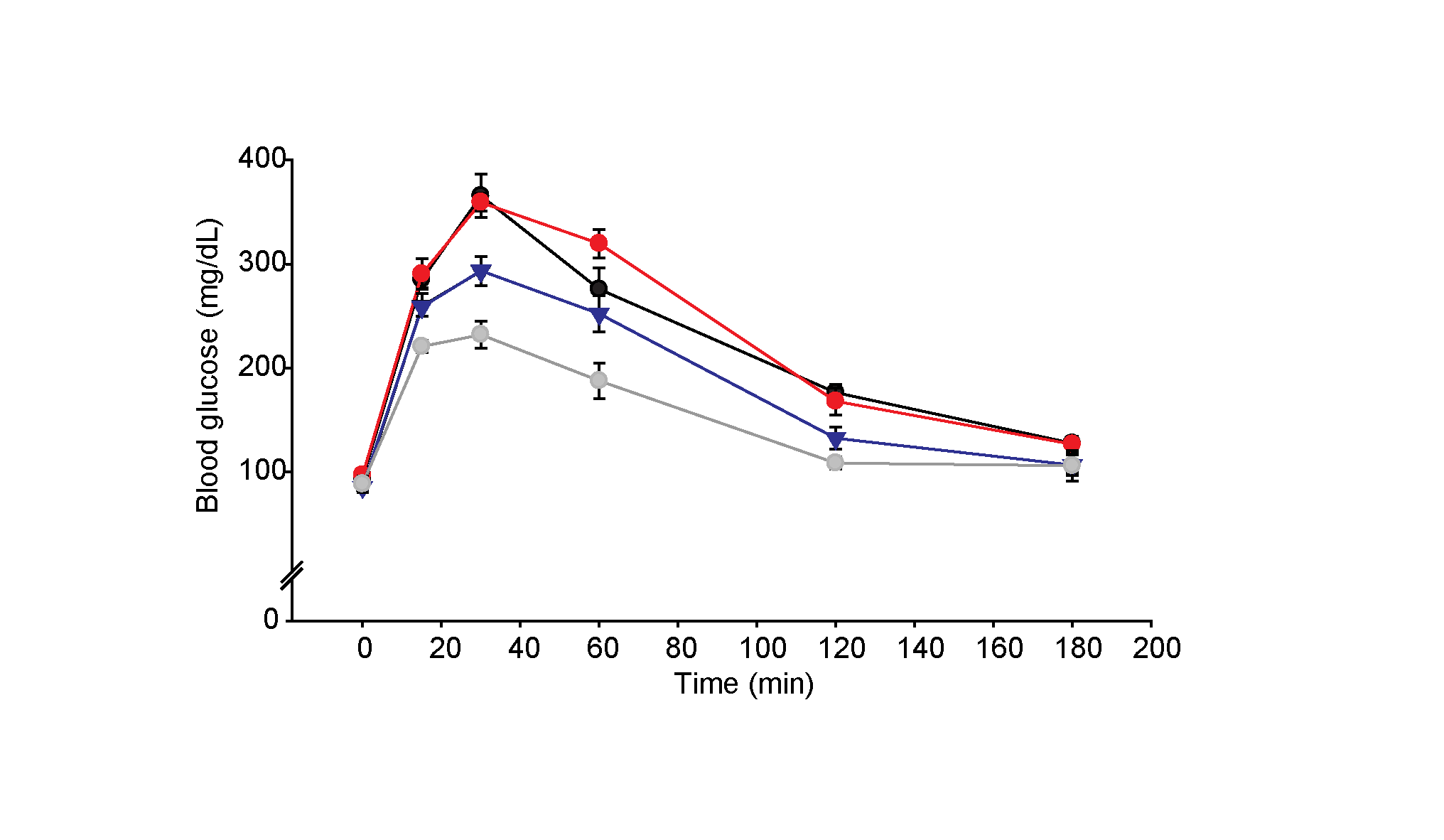

Supplement: Figure S1 — Glucose tolerance in response to dietary LC n -3 PUFA administered to mice in the ‘prevention study’. Mice were fed for 9 weeks a corn oil-based high-fat (cHF; black circles) diet, or experimental cHF+ω3TG (red circles) and cHF+ω3PL (blue triangles) diets containing 30 g DHA/EPA per kg diet. Some mice were also fed a low-fat Chow diet (grey circles). Plasma glucose profiles during 180 min following i.p. injection of glucose (time 0). Data are means±SEM. (TIF) [file pone.0038834.s001.tif]

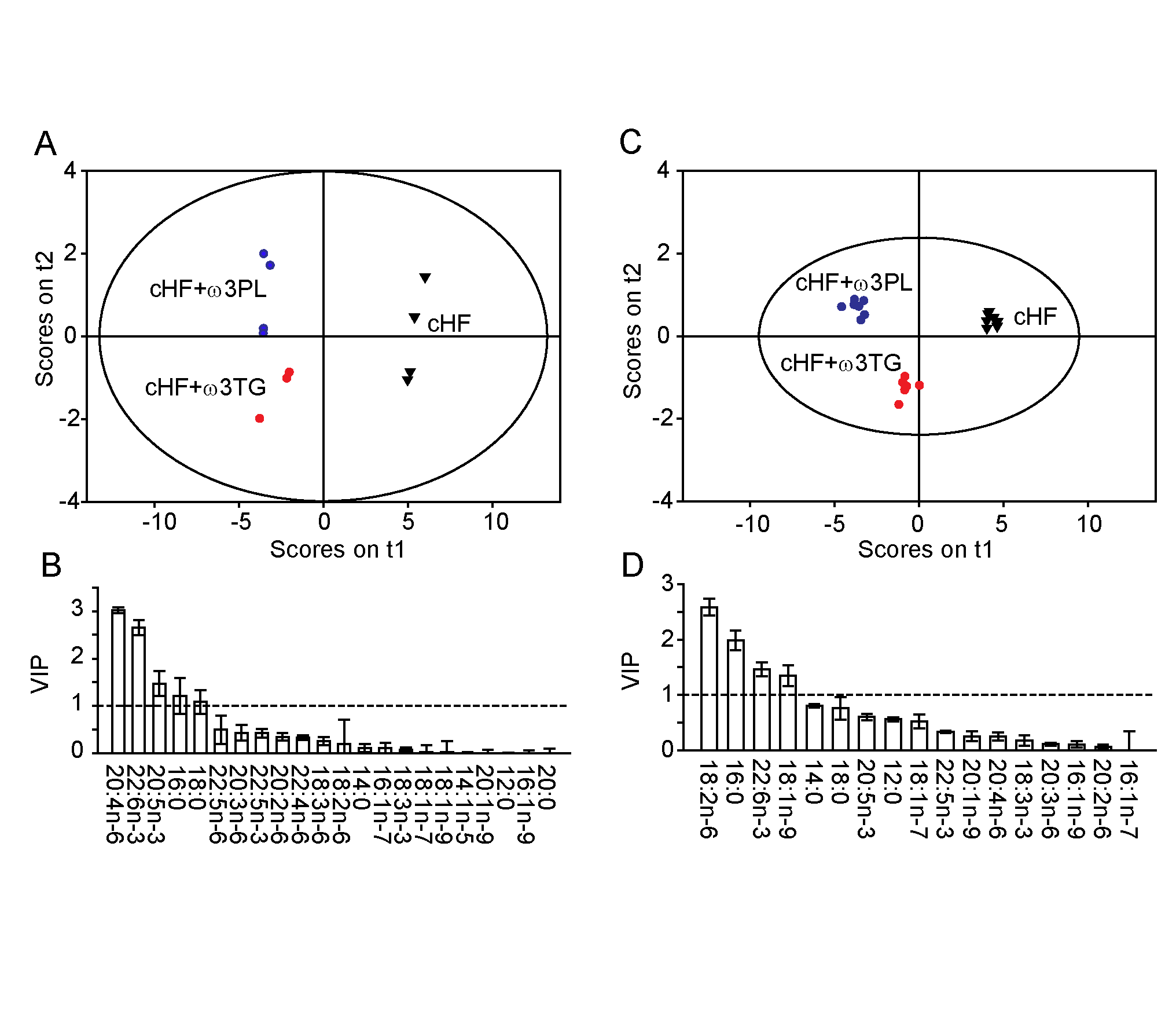

Supplement: Figure S2 — Orthogonal Partial Least Squares - Discriminant Analysis (Opls-DA) of fatty acid composition data in the liver and adipose tissue of mice in the ‘prevention study’. Mice were fed for 9 weeks a corn oil-based high-fat (cHF; black triangles) diet or cHF-based experimental diets, in which part of dietary lipids was replaced by LC n-3 PUFA concentrates either in the form of triglycerides (cHF+ω3TG; red circles) or marine phospholipids (cHF+ω3PL; blue circles) to achieve dietary EPA and DHA concentration of 30 g per kg diet. In total, 22 and 17 fatty acids were quantified in the phospholipid fraction from the liver and in total lipids from adipose tissue, respectively, using gas chromatography (see also Tables S6 and S7). To identify the major fatty acids discriminating between the cHF+ω3TG and cHF+ω3PL groups, multivariate analysis was performed on fatty acid profiles in liver phospholipids (A,B) and total lipids in adipose tissue (C,D) using the oPLS-DA algorithm. Within each tissue, mice (n = 4−7) were separated into 3 distinct groups based on the diet (A,C), and variables important to the projection of the first latent variable were plotted (B,D). To identify the most important ones, only variables with VIP scores greater than 1 (denoted by a solid horizontal line; B and D) and narrow confidence intervals were used for further evaluations (interpretations). (TIF) [file pone.0038834.s002.tif]

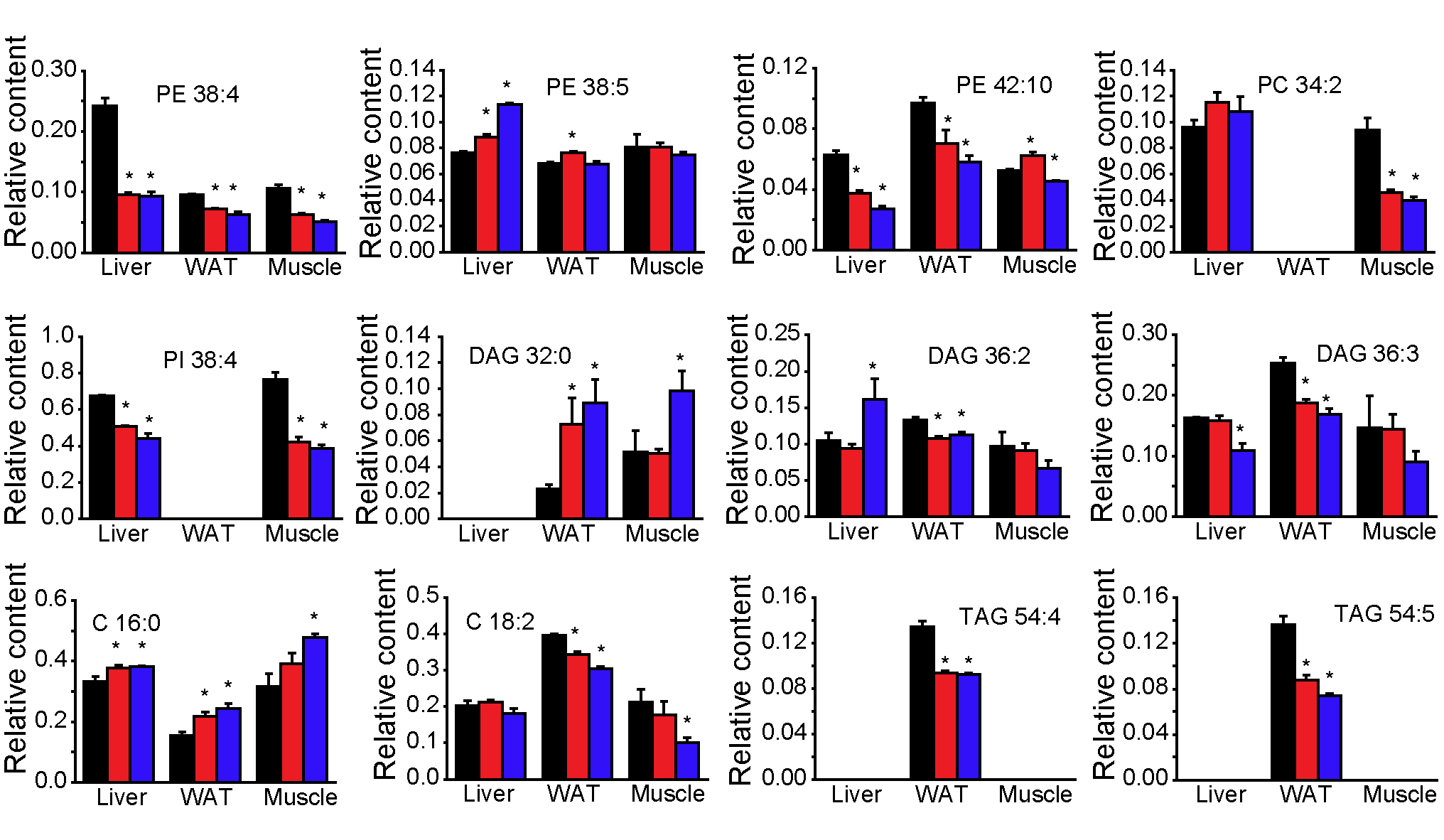

Supplement: Figure S3 — The levels of lipid species discriminating betweeen the phospholipid and triglyceride LC n −3 PUFA supplementation in the liver, adipose tissue and muscle of mice in the ‘prevention study’. Mice were fed for 9 weeks a corn oil-based high-fat (cHF; black bars) diet or cHF-based experimental diets, in which part of dietary lipids was replaced by LC n−3 PUFA concentrates either in the form of triglycerides (cHF+ω3TG; red bars) or phospholipids (cHF+ω3PL; blue bars) to achieve dietary EPA and DHA concentration of 30 g per kg diet. In total, 59, 71 and 61 lipid species were quantified in the liver, adipose tissue and skeletal muscle, respectively, using the TOF-SIMS analysis (see also Tables S8−S10). Major lipid species discriminating between the cHF+ω3TG and cHF+ω3PL groups were identified by the oPLS-DA algorithm. (TIF) [file pone.0038834.s003.tif]
