# Supplementary material for: Metabolic Effects of n-3 PUFA as Phospholipids Are Superior to Triglycerides in Mice Fed a High-Fat Diet: Possible Role of Endocannabinoids
Source: PLoS One. 2012 Jun 11;7(6):e38834. doi: 10.1371/journal.pone.0038834 (PMC3372498; doi:10.1371/journal.pone.0038834)
Supplement: Table S1 — Diets and treatments. cHF-based experimental diets were supplemented with the EPA and DHA concentrates either in the form of triglycerides (cHF+ω3TG diet) or marine phospholipids (cHF+ω3PL diet) to achieve various dietary EPA and DHA concentrations. In the ‘reversal study‘, all diets were also supplemented by 2 g metformin per kg diet. The concentrates were dissolved in corn oil prior to the preparation of experimental diets. aPercentage of dietary lipids replaced by EPAX 1050 TG (containing 46% DHA and 14% EPA). bPercentage of dietary lipids replaced by marine phospholipids (containing 20% DHA and 8% EPA). cPercentage of dietary lipids replaced by EPAX 1050 TG (containing 46% DHA and 14% EPA). dPercentage of dietary lipids replaced by marine phospholipids (containing 17% DHA and 5% EPA). See Table S2 for further details on the composition of marine phospholipids. -, not used in the respective study. (DOC) [file pone.0038834.s004.doc]

**Table S1** Diets and treatments

|  | Control diets |  | Experimental diets | | | |
| --- | --- | --- | --- | --- | --- | --- |
|  | cHF |  | cHF+ω3TG |  | cHF+ω3PL | |
| DHA/EPA (g per kg diet) | - |  | 30 |  | 10 | 30 |
| Macronutrient composition |  |  |  |  |  |  |
| Lipids (% diet, wt/wt) | 35.2 |  | 35.2 |  | 35.2 | 35.2 |
| Carbohydrates (% diet, wt/wt) | 35.4 |  | 35.4 |  | 35.4 | 35.4 |
| Proteins (% diet, wt/wt) | 20.5 |  | 20.5 |  | 20.5 | 20.5 |
| Energy density (kJ/g) | 22.8 |  | 22.8 |  | 22.8 | 22.8 |
| Supplements – ‘prevention study’ |  |  |  |  |  |  |
| EPAX 1050 TG (%)^a^ | 0 |  | 15 |  | 0 | 0 |
| Marine phospholipids (%)^b^ | 0 |  | 0 |  | 11 | 33 |
| Supplements – ‘reversal study’ |  |  |  |  |  |  |
| EPAX 1050 TG (%)^c^ | 0 |  | 15 |  | - | 0 |
| Marine phospholipids (%)^d^ | 0 |  | 0 |  | - | 41 |

cHF-based experimental diets were supplemented with the EPA and DHA concentrates either in the form of triglycerides (cHF+ω3TG diet) or marine phospholipids (cHF+ω3PL diet) to achieve various dietary EPA and DHA concentrations. In the **‘**reversal study‘, all diets were also supplemented by 2 g metformin per kg diet. The concentrates were dissolved in corn oil prior to the preparation of experimental diets.

^a^Percentage of dietary lipids replaced by EPAX 1050 TG (containing 46 % DHA and 14 % EPA).

^b^Percentage of dietary lipids replaced by marine phospholipids (containing 20 % DHA and 8 % EPA).

^c^Percentage of dietary lipids replaced by EPAX 1050 TG (containing 46 % DHA and 14 % EPA).

^d^Percentage of dietary lipids replaced by marine phospholipids (containing 17 % DHA and 5 % EPA).

See Table S2 for further details on the composition of marine phospholipids.

-, not used in the respective study.
