# Supplementary material for: Metabolic Effects of n-3 PUFA as Phospholipids Are Superior to Triglycerides in Mice Fed a High-Fat Diet: Possible Role of Endocannabinoids
Source: PLoS One. 2012 Jun 11;7(6):e38834. doi: 10.1371/journal.pone.0038834 (PMC3372498; doi:10.1371/journal.pone.0038834)
Supplement: Table S2 — Phospholipid and fatty acid composition of the EPA and DHA concentrate based on marine phospholipids. Food grade herring meal was extracted once with ethanol (ethanol/meal = 5∶1 vol/wt) at 60°C. The extract was concentrated to dryness in vacuo on a rotary evaporator. The resulting residue was treated with ice cold acetone to extract the bulk of neutral lipids from the marine phospholipid concentrate with the EPA and DHA content of 50−80 and 170−200 mg per g diet, respectively. Total phospholipid content in the marine phospholipid concentrate was 74−80% (of three different analyses), while the content of major phospholipid fractions in the concentrate was as follows: phosphatidylcholine (PC; 47−56%), lysoPC (∼3%), phosphatidylethanolamine (PE; 9−13), lysoPE (2−3%). Other minor phospholipid fractions in the product include phosphatidylinositol (∼1%), sphingosine (2−4%), and unidentified fraction (2–9%). Fatty acid composition (mol %) of a typical batch determined by gas chromatography is shown. MUFA, monounsaturated fatty acids. –, ≤0.1% (detection limit). (DOC) [file pone.0038834.s005.doc]

**Table S2** Phospholipid and fatty acid composition of the EPA and DHA concentrate based on marine phospholipids

| Fatty acids | PC | lysoPC | PE | lysoPE | Total lipids in the product |
| --- | --- | --- | --- | --- | --- |
| *Saturated* |  |  |  |  |  |
| 12:0 | - | 0.18 | 0.82 | 0.20 | - |
| 14:0 | 4.04 | 4.68 | 2.56 | 2.02 | 2.59 |
| 16:0 | 31.50 | 49.29 | 26.83 | 30.30 | 25.61 |
| 18:0 | 1.07 | 2.16 | 3.80 | 4.87 | 1.12 |
| 20:0 | - | - | 0.15 | 0.17 | - |
| 22:0 | - | - | - | - | 0.54 |
| Total | 36.61 | 56.31 | 34.16 | 37.56 | 29.92 |
| *MUFA* |  |  |  |  |  |
| 16:1 *n*-9 | 0.18 | 0.30 | 0.51 | 0.16 | - |
| 16:1 *n*-7 | 2.22 | 2.39 | 2.23 | 2.27 | 2.13 |
| 18:1 trans | - | 0.14 | 0.14 | - | - |
| 18:1 *n*-9 | 4.89 | 5.25 | 8.88 | 10.10 | 5.34 |
| 18:1 *n*-7 | 2.70 | 2.98 | 4.76 | 7.80 | 3.31 |
| 20:1 *n*-9 | 1.09 | 1.44 | 4.31 | 5.39 | 1.28 |
| Total | 11.08 | 12.50 | 20.83 | 25.72 | 12.08 |
| *n-6 PUFA* |  |  |  |  |  |
| 18:2 *n*-6 | 1.27 | 1.36 | 1.94 | 2.69 | 1.13 |
| 20:2 *n*-6 | 0.22 | 0.29 | 0.44 | 0.43 | 0.11 |
| 20:4 *n*-6 | - | - | - | - | 0.61 |
| Total | 1.49 | 1.65 | 2.38 | 3.12 | 1.92 |
| *n-3 PUFA* |  |  |  |  |  |
| 18:3 *n*-3 | 0.69 | 0.70 | 0.97 | 0.89 | 0.36 |
| 20:3 *n*-3 | 0.92 | 0.62 | 1.34 | 0.91 | - |
| 20:4 *n*-3 | 0.60 | 0.60 | 1.13 | 0.59 | - |
| 20:5 *n*-3 | 14.46 | 8.39 | 7.48 | 4.39 | 14.51 |
| 22:3 *n*-3 | 0.21 | 0.09 | 0.16 | 0.08 | - |
| 22:4 *n*-3 | 0.28 | 0.15 | 0.49 | 0.36 | - |
| 22:5 *n*-3 | 0.91 | 0.52 | 0.88 | 0.64 | 0.84 |
| 22:6 *n*-3 | 32.60 | 18.31 | 30.02 | 25.61 | 39.99 |
| Total | 50.67 | 29.38 | 42.47 | 33.47 | 56.09 |
|  |  |  |  |  |  |

Food grade herring meal was extracted once with ethanol (ethanol/meal = 5:1 vol/wt) at 60°C. The extract was concentrated to dryness in vacuo on a rotary evaporator. The resulting residue was treated with ice cold acetone to extract the bulk of neutral lipids from the marine phospholipid concentrate with the EPA and DHA content of 50-80 and 170-200 mg per g diet, respectively. Total phospholipid content in the marine phospholipid concentrate was 74-80 % (of three different analyses), while the content of major phospholipid fractions in the concentrate was as follows: phosphatidylcholine (PC; 47-56 %), lysoPC (~3 %), phosphatidylethanolamine (PE; 9-13), lysoPE (2-3 %). Other minor phospholipid fractions in the product include phosphatidylinositol (~1 %), sphingosine (2-4 %), and unidentified fraction (2-9 %). Fatty acid composition (mol %) of a typical batch determined by gas chromatography is shown. MUFA, monounsaturated fatty acids. – , ≤0.1 % (detection limit).
