# Supplementary material for: Metabolic Effects of n-3 PUFA as Phospholipids Are Superior to Triglycerides in Mice Fed a High-Fat Diet: Possible Role of Endocannabinoids
Source: PLoS One. 2012 Jun 11;7(6):e38834. doi: 10.1371/journal.pone.0038834 (PMC3372498; doi:10.1371/journal.pone.0038834)
Supplement: Table S3 — Fatty acid composition of experimental diets in the ‘prevention study’. cHF-based experimental diets were supplemented with the EPA and DHA concentrates either in the form of triglycerides (cHF+ω3TG diet) or marine phospholipids (cHF+ω3PL diet) to achieve various dietary EPA and DHA concentrations. Fatty acid composition (mol %) was analyzed in triplicates in the total lipid fraction extracted from the experimental diets. Standard errors are not shown, but for most fatty acids represented <5% of the mean. SFA, saturated fatty acids; MUFA, monounsaturated fatty acids; PUFA, polyunsaturated fatty acids. –, ≤0.1% (detection limit). (DOC) [file pone.0038834.s006.doc]

**Table S3** Fatty acid composition of experimental diets in the ‘prevention study’

|  | cHF |  | cHF+ω3TG |  | cHF+ω3PL | |
| --- | --- | --- | --- | --- | --- | --- |
| DHA/EPA (g per kg diet) | 0 |  | 30 |  | 10 | 30 |
| *Saturated* |  |  |  |  |  |  |
| 8:0 | 0.12 |  | 0.20 |  | - | - |
| 10:0 | 0.35 |  | 0.48 |  | 0.22 | 0.36 |
| 12:0 | 1.66 |  | 1.89 |  | 1.50 | 1.90 |
| 14:0 | 1.90 |  | 2.09 |  | 2.01 | 2.74 |
| 16:0 | 13.47 |  | 12.82 |  | 14.40 | 16.98 |
| 18:0 | 2.69 |  | 2.75 |  | 2.65 | 2.74 |
| 20:0 | 0.28 |  | 0.30 |  | 0.26 | 0.21 |
| Total | 20.47 |  | 20.53 |  | 21.05 | 24.99 |
|  |  |  |  |  |  |  |
| *MUFA* |  |  |  |  |  |  |
| 14:1 *n*-9 | - |  | 0.11 |  | 0.10 | 0.11 |
| 16:1 *n*-7 | 0.27 |  | 0.41 |  | 0.38 | 0.71 |
| 18:1 *n*-9 | 27.30 |  | 24.54 |  | 26.34 | 23.39 |
| 18:1 *n*-7 | 0.81 |  | 0.88 |  | 1.02 | 1.43 |
| 20:1 *n*-9 | 0.21 |  | 0.34 |  | 0.29 | 0.47 |
| Total | 28.72 |  | 26.32 |  | 28.18 | 26.19 |
|  |  |  |  |  |  |  |
| *n*-6 *PUFA* |  |  |  |  |  |  |
| 18:2 *n*-6 | 49.40 |  | 41.40 |  | 46.20 | 36.00 |
| 20:4 *n*-6 | - |  | 0.18 |  | - | 0.16 |
| Total | 49.36 |  | 41.61 |  | 46.26 | 36.22 |
|  |  |  |  |  |  |  |
| *n*-3 *PUFA* |  |  |  |  |  |  |
| 18:3 *n*-3 | 1.45 |  | 1.37 |  | 1.44 | 1.36 |
| 20:4 *n*-3 | - |  | 0.10 |  | - | - |
| 20:5 *n*-3 | - |  | 2.15 |  | 0.82 | 2.93 |
| 22:5 *n*-3 | - |  | 0.53 |  | - | 0.20 |
| 22:6 *n*-3 | - |  | 7.36 |  | 2.14 | 8.00 |
| Total | 1.45 |  | 11.54 |  | 4.51 | 12.60 |
|  |  |  |  |  |  |  |
| *n*-6 PUFA / *n*-3 PUFA | 34.0 : 1 |  | 3.6 : 1 |  | 10.3 : 1 | 2.9 : 1 |
|  |  |  |  |  |  |  |

cHF-based experimental diets were supplemented with the EPA and DHA concentrates either in the form of triglycerides (cHF+ω3TG diet) or marine phospholipids (cHF+ω3PL diet) to achieve various dietary EPA and DHA concentrations. Fatty acid composition (mol %) was analyzed in triplicates in the total lipid fraction extracted from the experimental diets. Standard errors are not shown, but for most fatty acids represented <5 % of the mean. SFA, saturated fatty acids; MUFA, monounsaturated fatty acids; PUFA, polyunsaturated fatty acids. – , ≤0.1 % (detection limit).
