# Supplementary material for: Metabolic Effects of n-3 PUFA as Phospholipids Are Superior to Triglycerides in Mice Fed a High-Fat Diet: Possible Role of Endocannabinoids
Source: PLoS One. 2012 Jun 11;7(6):e38834. doi: 10.1371/journal.pone.0038834 (PMC3372498; doi:10.1371/journal.pone.0038834)
Supplement: Table S4 — Fatty acid composition of plasma in the ‘prevention study’. Fatty acid composition was analyzed in the total lipid fraction extracted from plasma. The results (mol %) are expressed as means ± SEM (n = 4). a,b,cSignificant differences (ANOVA) compared with cHF, cHF+ω3TG, and cHF+ω3PL (10 g per kg diet), respectively. Similar results were obtained when fatty acid composition in plasma was analyzed already after 2 weeks of dietary intervention (not shown). MUFA, monounsaturated fatty acids; PUFA, polyunsaturated fatty acids. –, ≤0.1% (detection limit). (DOC) [file pone.0038834.s007.doc]

**Table S4** Fatty acid composition of plasma in the ‘prevention study’

|  | cHF |  | cHF+ω3TG |  | cHF+ω3PL | |
| --- | --- | --- | --- | --- | --- | --- |
| DHA/EPA (g per kg diet) | 0 |  | 30 |  | 10 | 30 |
| *Saturated* |  |  |  |  |  |  |
| 12:0 | 0.26 ± 0.04 |  | 0.25 ± 0.09 |  | 0.19 ± 0.02 | 0.16 ± 0.01 |
| 14:0 | 0.58 ± 0.06 |  | 0.48 ± 0.04 |  | 0.75 ± 0.10 | 0.66 ± 0.05 |
| 16:0 | 18.14 ± 0.29 |  | 18.42 ± 0.44 |  | 19.29 ± 0.27 | 18.23 ± 0.16 |
| 18:0 | 12.16 ± 0.57 |  | 10.11 ± 0.20 |  | 10.52 ± 1.11 | 8.52 ± 0.22^a^ |
| 20:0 | 0.20 ± 0.03 |  | 0.20 ± 0.03 |  | 0.25 ± 0.01 | 0.16 ± 0.01 |
| Total | 31.34 ± 0.51 |  | 29.47 ± 0.41 |  | 31.01 ± 1.48 | 27.73 ± 0.29^a^ |
|  |  |  |  |  |  |  |
| *MUFA* |  |  |  |  |  |  |
| 16:1 *n*-7 | 0.24 ± 0.02 |  | 0.25 ± 0.01 |  | 0.30 ± 0.02^ab^ | 0.41 ± 0.01^abc^ |
| 18:1 *n*-9 | 8.92 ± 0.44 |  | 8.33 ± 0.17 |  | 9.26 ± 0.27 | 7.75 ± 0.60 |
| 18:1 *n*-7 | 0.84 ± 0.07 |  | 0.82 ± 0.25 |  | 0.90 ± 0.07 | 0.97 ± 0.06 |
| 20:1 *n*-9 | 0.23 ± 0.02 |  | 0.22 ± 0.00 |  | 0.32 ± 0.01 | 0.36 ± 0.01^ab^ |
| Total | 10.23 ± 0.47 |  | 9.62 ± 0.39 |  | 10.78 ± 0.35 | 9.49 ± 0.68 |
|  |  |  |  |  |  |  |
| *n-6 PUFA* |  |  |  |  |  |  |
| 18:2 *n*-6 | 41.82 ± 0.71 |  | 40.35 ± 0.45 |  | 42.67 ± 1.07 | 36.07 ± 0.65^abc^ |
| 18:3 *n*-6 | 0.31 ± 0.02 |  | 0.13 ± 0.02^a^ |  | 0.18 ± 0.03^a^ | 0.13 ± 0.02^a^ |
| 20:2 *n*-6 | 0.18 ± 0.01 |  | 0.14 ± 0.02 |  | 0.14 ± 0.01 | 0.11 ± 0.01^a^ |
| 20:3 *n*-6 | 0.68 ± 0.06 |  | 0.54 ± 0.06 |  | 0.67 ± 0.06 | 0.49 ± 0.04 |
| 20:4 *n*-6 | 12.17 ± 0.50 |  | 3.84 ± 0.22^a^ |  | 3.48 ± 0.24^a^ | 4.08 ± 0.21^a^ |
| Total | 55.17 ± 0.72 |  | 45.01 ± 0.16^a^ |  | 47.14 ± 1.36 | 40.88 ± 0.82^abc^ |
|  |  |  |  |  |  |  |
| *n-3 PUFA* |  |  |  |  |  |  |
| 18:3 *n*-3 | 0.29 ± 0.04 |  | 0.28 ± 0.03 |  | 0.34 ± 0.03 | 0.25 ± 0.02 |
| 20:5 *n*-3 (EPA) | - |  | 4.80 ± 0.22^a^ |  | 2.84 ± 0.05^ab^ | 9.47 ± 0.33^abc^ |
| 22:5 *n*-3 | 0.28 ± 0.04 |  | 0.32 ± 0.04 |  | 0.48 ± 0.05^a^ | 0.33 ± 0.01 |
| 22:6 *n*-3 (DHA) | 2.60 ± 0.17 |  | 10.51 ± 0.31^a^ |  | 7.41 ± 0.26^ab^ | 11.86 ± 0.34^abc^ |
| Total | 3.26 ± 0.18 |  | 15.91 ± 0.34^a^ |  | 11.08 ± 0.22 | 21.91 ± 0.07^a^ |
| Sum EPA+DHA | 2.68 ± 0.17 |  | 15.31 ± 0.31^a^ |  | 10.26 ± 0.24^ab^ | 21.33 ± 0.09^abc^ |
|  |  |  |  |  |  |  |

Fatty acid composition was analyzed in the total lipid fraction extracted from plasma. The results (mol %) are expressed as means ± SEM (*n*=4).

^a,b,c^Significant differences (ANOVA) compared with cHF, cHF+ω3TG, and cHF+ω3PL (10 g per kg diet), respectively. Similar results were obtained when fatty acid composition in plasma was analyzed already after 2 weeks of dietary intervention (not shown). MUFA, monounsaturated fatty acids; PUFA, polyunsaturated fatty acids. – , ≤0.1 % (detection limit).
