# Supplementary material for: Metabolic Effects of n-3 PUFA as Phospholipids Are Superior to Triglycerides in Mice Fed a High-Fat Diet: Possible Role of Endocannabinoids
Source: PLoS One. 2012 Jun 11;7(6):e38834. doi: 10.1371/journal.pone.0038834 (PMC3372498; doi:10.1371/journal.pone.0038834)
Supplement: Table S5 — Fatty acid composition of total triglycerides in the liver from the ‘prevention study’. Fatty acid composition was analyzed in the triglyceride fraction extracted from the liver. The results (mol %) are expressed as means ± SEM (n = 4). a,b,cSignificant differences (ANOVA) compared with cHF, cHF+ω3TG, and cHF+ω3PL (10 g per kg diet), respectively. SFA, saturated fatty acids; MUFA, monounsaturated fatty acids; PUFA, polyunsaturated fatty acids. –, ≤0.1% (detection limit). (DOC) [file pone.0038834.s008.doc]

**Table S5** Fatty acid composition of total triglycerides in the liver from the ‘prevention study’

|  | cHF |  | cHF+ω3TG |  | cHF+ω3PL | |
| --- | --- | --- | --- | --- | --- | --- |
| DHA/EPA (g per kg diet) | 0 |  | 30 |  | 10 | 30 |
| *SFA* |  |  |  |  |  |  |
| 12:0 | 0.16 ± 0.02 |  | 0.14 ± 0.03 |  | 0.29 ± 0.06 | 0.20 ± 0.03 |
| 14:0 | 0.64 ± 0.04 |  | 0.69 ± 0.05 |  | 0.74 ± 0.12 | 0.97 ± 0.08^a^ |
| 16:0 | 18.69 ± 0.94 |  | 17.86 ± 2.03 |  | 20.49 ± 0.90 | 19.01 ± 0.73 |
| 18:0 | 1.98 ± 0.18 |  | 2.03 ± 0.31 |  | 2.39 ± 0.30 | 2.76 ± 0.21 |
| 20:0 | 0.19 ± 0.01 |  | 0.14 ± 0.03 |  | 0.19 ± 0.01 | 0.19 ± 0.02 |
| Total | 21.66 ± 0.95 |  | 20.85 ± 2.03 |  | 24.09 ± 0.61 | 23.13 ± 0.67 |
|  |  |  |  |  |  |  |
| *MUFA* |  |  |  |  |  |  |
| 16:1 *n*-9 | 0.65 ± 0.08 |  | 0.70 ± 0.12 |  | 0.67 ± 0.07 | 0.43 ± 0.03 |
| 16:1 *n*-7 | 0.66 ± 0.04 |  | 0.81 ± 0.26 |  | 0.61 ± 0.04 | 0.78 ± 0.02 |
| 18:1 *n*-9 | 23.19 ± 0.84 |  | 18.95 ± 1.59 |  | 21.66 ± 1.10 | 22.21 ± 0.91 |
| 18:1 *n*-7 | 0.81 ± 0.06 |  | 0.72 ± 0.21 |  | 0.76 ± 0.08 | 1.03 ± 0.10 |
| 20:1 *n*-9 | 0.49 ± 0.04 |  | 0.33 ± 0.04^a^ |  | 0.43 ± 0.03 | 0.43 ± 0.03 |
| Total | 25.82 ± 0.90 |  | 21.53 ± 2.11 |  | 24.17 ± 1.18 | 24.90 ± 1.02 |
|  |  |  |  |  |  |  |
| *n-6 PUFA* |  |  |  |  |  |  |
| 18:2 *n*-6 | 45.28 ± 0.60 |  | 34.37 ± 1.29^a^ |  | 40.47 ± 1.04^ab^ | 31.76 ± 0.26^ac^ |
| 20:2 *n*-6 | 0.57 ± 0.08 |  | 0.18 ± 0.04^a^ |  | 0.26 ± 0.03^a^ | 0.18 ± 0.01^a^ |
| 18:3 *n*-6 | 0.79 ± 0.05 |  | 0.22 ± 0.01^a^ |  | 0.35 ± 0.05 | 0.16 ± 0.01^a^ |
| 20:3 *n*-6 | 1.12 ± 0.13 |  | 0.39 ± 0.06^a^ |  | 0.54 ± 0.04^a^ | 0.34 ± 0.02^ac^ |
| 20:4 *n*-6 | 1.89 ± 0.12 |  | 0.59 ± 0.14^a^ |  | 0.60 ± 0.06^a^ | 0.36 ± 0.04^a^ |
| 22:4 *n*-6 | 0.43 ± 0.06 |  | - |  | - | - |
| 22:5 *n*-6 | 0.51 ± 0.14 |  | 0.17 ± 0.02 |  | - | - |
| Total | 50.61 ± 0.35 |  | 35.97 ± 1.21^a^ |  | 42.33 ± 1.07^ab^ | 32.88 ± 0.20^abc^ |
|  |  |  |  |  |  |  |
| *n-3 PUFA* |  |  |  |  |  |  |
| 18:3 *n*-3 | 0.75 ± 0.07 |  | 0.85 ± 0.04 |  | 0.75 ± 0.04 | 0.70 ± 0.04 |
| 20:5 *n*-3 (EPA) | - |  | 2.37 ± 0.19^a^ |  | 0.93 ± 0.04 | 1.98 ± 0.05^a^ |
| 22:5 *n*-3 | 0.21 ± 0.02 |  | 1.54 ± 0.12^a^ |  | 0.72 ± 0.03^ab^ | 1.15 ± 0.03^abc^ |
| 22:6 *n*-3 (DHA) | 0.89 ± 0.01 |  | 16.89 ± 3.02^a^ |  | 7.01 ± 0.37 | 15.26 ± 0.77^a^ |
| Total | 1.92 ± 0.10 |  | 21.65 ± 3.00^a^ |  | 9.41 ± 0.46^ab^ | 19.09 ± 0.87^ac^ |
| Sum EPA+DHA | 0.97 ± 0.02 |  | 19.26 ± 2.91^a^ |  | 7.94 ± 0.41^ab^ | 17.24 ± 0.81^ac^ |
|  |  |  |  |  |  |  |

Fatty acid composition was analyzed in the triglyceride fraction extracted from the liver. The results (mol %) are expressed as means ± SEM (*n*=4).

^a,b,c^Significant differences (ANOVA) compared with cHF, cHF+ω3TG, and cHF+ω3PL (10 g per kg diet), respectively. SFA, saturated fatty acids; MUFA, monounsaturated fatty acids; PUFA, polyunsaturated fatty acids. – , ≤0.1 % (detection limit).
