# Supplementary material for: Metabolic Effects of n-3 PUFA as Phospholipids Are Superior to Triglycerides in Mice Fed a High-Fat Diet: Possible Role of Endocannabinoids
Source: PLoS One. 2012 Jun 11;7(6):e38834. doi: 10.1371/journal.pone.0038834 (PMC3372498; doi:10.1371/journal.pone.0038834)
Supplement: Table S6 — Fatty acid composition of total phospholipids in the liver from the ‘prevention study’. Fatty acid composition was analyzed in the total phospholipid fraction extracted from the liver. The results (mol %) are expressed as means ± SEM (n = 4). a,b,cSignificant differences (ANOVA) compared with cHF, cHF+ω3TG, and cHF+ω3PL (10 g per kg diet), respectively. SFA, saturated fatty acids; MUFA, monounsaturated fatty acids; PUFA, polyunsaturated fatty acids. –, ≤0.1% (detection limit). (DOC) [file pone.0038834.s009.doc]

**Table S6** Fatty acid composition of total phospholipids in the liver from the ‘prevention study’

|  | cHF |  | cHF+ω3TG |  | cHF+ω3PL | |
| --- | --- | --- | --- | --- | --- | --- |
| DHA/EPA (g/kg diet) | 0 |  | 30 |  | 10 | 30 |
| *SFA* |  |  |  |  |  |  |
| 14:0 | - |  | 0.10 ± 0.01 |  | 0.12 ± 0.00^ab^ | 0.13 ± 0.01^ab^ |
| 16:0 | 21.13 ± 0.44 |  | 24.14 ± 1.64 |  | 25.33 ± 0.65 | 24.30 ± 0.55 |
| 18:0 | 16.94 ± 0.15 |  | 15.58 ± 0.83 |  | 13.42 ± 0.35^ab^ | 14.27 ± 0.29^a^ |
| 20:0 | 0.21 ± 0.04 |  | 0.22 ± 0.05 |  | 0.25 ± 0.03 | 0.19 ± 0.02 |
| Total | 38.37 ± 0.52 |  | 40.04 ± 0.84 |  | 39.12 ± 0.50 | 38.90 ± 0.79 |
|  |  |  |  |  |  |  |
| *MUFA* |  |  |  |  |  |  |
| 16:1 *n*-9 | 0.11 ± 0.01 |  | 0.11 ± 0.01 |  | 0.13 ± 0.01 | 0.10 ± 0.00 |
| 16:1 *n*-7 | 0.16 ± 0.02 |  | 0.16 ± 0.01 |  | 0.16 ± 0.01 | 0.21 ± 0.01^ab^ |
| 18:1 *n*-9 | 6.25 ± 0.40 |  | 5.60 ± 0.32 |  | 6.18 ± 0.53 | 6.66 ± 0.50 |
| 18:1 *n*-7 | 0.65 ± 0.05 |  | 0.54 ± 0.14 |  | 0.54 ± 0.05 | 0.75 ± 0.08 |
| 20:1 *n*-9 | 0.20 ± 0.00 |  | 0.17 ± 0.01 |  | 0.21 ± 0.01 | 0.21 ± 0.02 |
| Total | 7.37 ± 0.44 |  | 6.58 ± 0.43 |  | 7.23 ± 0.59 | 7.94 ± 0.60 |
|  |  |  |  |  |  |  |
| *n-6 PUFA* |  |  |  |  |  |  |
| 18:2 *n*-6 | 21.78 ± 0.35 |  | 21.18 ± 0.81 |  | 25.36 ± 0.25^b^ | 21.26 ± 0.24^c^ |
| 20:2 *n*-6 | 0.44 ± 0.03 |  | 0.27 ± 0.02^a^ |  | 0.30 ± 0.01^a^ | 0.20 ± 0.01^ac^ |
| 18:3 *n*-6 | 0.19 ± 0.02 |  | - |  | 0.15 ± 0.01^b^ | - |
| 20:3 *n*-6 | 1.48 ± 0.06 |  | 1.23 ± 0.15 |  | 1.98 ± 0.01 | 1.14 ± 0.02 |
| 20:4 *n*-6 | 19.99 ± 0.47 |  | 5.89 ± 0.55^a^ |  | 6.94 ± 0.28^a^ | 4.40 ± 0.08^abc^ |
| 22:4 *n*-6 | 0.20 ± 0.02 |  | - |  | - | - |
| 22:5 *n*-6 | 0.59 ± 0.17 |  | 0.16 ± 0.01 |  | - | - |
| Total | 44.67 ± 0.28 |  | 28.83 ± 0.46^a^ |  | 34.77 ± 0.16^ab^ | 27.12 ± 0.25^abc^ |
|  |  |  |  |  |  |  |
| *n-3 PUFA* |  |  |  |  |  |  |
| 20:5 *n*-3 (EPA) | - |  | 2.83 ± 0.18^a^ |  | 1.54 ± 0.09 | 4.17 ± 0.22^a^ |
| 22:5 *n*-3 | 0.19 ± 0.01 |  | 0.48 ± 0.03^a^ |  | 0.27 ± 0.04^b^ | 0.54 ± 0.04^ac^ |
| 22:6 *n*-3 (DHA) | 9.28 ± 0.18 |  | 21.15 ± 0.53^a^ |  | 16.98 ± 0.30^ab^ | 21.25 ± 0.35^ac^ |
| Total | 9.59 ± 0.21 |  | 24.54 ± 0.53^a^ |  | 18.88 ± 0.31^ab^ | 26.04 ± 0.26^abc^ |
| Sum EPA+DHA | 9.33 ± 0.19 |  | 23.98 ± 0.50^a^ |  | 18.52 ± 0.31^ab^ | 25.42 ± 0.27^abc^ |
|  |  |  |  |  |  |  |

Fatty acid composition was analyzed in the total phospholipid fraction extracted from the liver. The results (mol %) are expressed as means ± SEM (*n*=4).

^a,b,c^Significant differences (ANOVA) compared with cHF, cHF+ω3TG, and cHF+ω3PL (10 g per kg diet), respectively. SFA, saturated fatty acids; MUFA, monounsaturated fatty acids; PUFA, polyunsaturated fatty acids. – , ≤0.1 % (detection limit).
