# Supplementary material for: Metabolic Effects of n-3 PUFA as Phospholipids Are Superior to Triglycerides in Mice Fed a High-Fat Diet: Possible Role of Endocannabinoids
Source: PLoS One. 2012 Jun 11;7(6):e38834. doi: 10.1371/journal.pone.0038834 (PMC3372498; doi:10.1371/journal.pone.0038834)
Supplement: Table S7 — Fatty acid composition in total lipids from adipose tissue in the ‘prevention study’. Fatty acid composition was analyzed in the total lipid fraction extracted from abdominal adipose tissue (epididymal fat depot). Results (mol %) are expressed as means ± SEM (n = 7). a,b,cSignificant differences (ANOVA) compared with cHF, cHF+ω3TG, and cHF+ω3PL (10 g per kg diet), respectively. MUFA, monounsaturated fatty acids; PUFA, polyunsaturated fatty acids. –, ≤0.1% (detection limit). (DOC) [file pone.0038834.s010.doc]

**Table S7** Fatty acid composition in total lipids from adipose tissue in the ‘prevention study’

|  | cHF |  | cHF+ω3TG |  | cHF+ω3PL | |
| --- | --- | --- | --- | --- | --- | --- |
| DHA/EPA (g/kg diet) | 0 |  | 30 |  | 10 | 30 |
| *Saturated* |  |  |  |  |  |  |
| 12:0 | 0.52 ± 0.02 |  | 0.88 ± 0.09^a^ |  | 0.67 ± 0.03^b^ | 1.07 ± 0.05^abc^ |
| 14:0 | 1.15 ± 0.02 |  | 1.72 ± 0.09^a^ |  | 1.51 ± 0.04^ab^ | 2.27 ± 0.06^abc^ |
| 16:0 | 13.25 ± 0.50 |  | 17.03 ± 0.39^a^ |  | 15.54 ± 0.37^a^ | 20.22 ± 0.67^abc^ |
| 18:0 | 1.26 ± 0.07 |  | 1.90 ± 0.12^a^ |  | 1.68 ± 0.14 | 2.34 ± 0.13^a^ |
| Total | 16.22 ± 0.53 |  | 21.57 ± 0.46^a^ |  | 19.44 ± 0.40^ab^ | 25.95 ± 0.80^abc^ |
|  |  |  |  |  |  |  |
| *MUFA* |  |  |  |  |  |  |
| 16:1 *n*-9 | 0.36 ± 0.02 |  | 0.39 ± 0.03 |  | 0.32 ± 0.02 | 0.29 ± 0.02^b^ |
| 16:1 *n*-7 | 2.48 ± 0.20 |  | 2.40 ± 0.46 |  | 3.04 ± 0.65 | 2.47 ± 0.43 |
| 18:1 *n*-9 | 32.60 ± 0.43 |  | 28.47 ± 0.45^a^ |  | 31.25 ± 0.33^b^ | 29.16 ± 0.36^ac^ |
| 18:1 *n*-7 | 1.14 ± 0.03 |  | 1.19 ± 0.04 |  | 1.37 ± 0.04^ab^ | 1.72 ± 0.05^abc^ |
| 20:1 *n*-9 | 0.30 ± 0.01 |  | 0.30 ± 0.01 |  | 0.32 ± 0.01 | 0.47 ± 0.04^abc^ |
| Total | 36.95 ± 0.42 |  | 32.85 ± 0.81^a^ |  | 36.39 ± 0.47^b^ | 34.22 ± 0.77^a^ |
|  |  |  |  |  |  |  |
| *n-6 PUFA* |  |  |  |  |  |  |
| 18:2 *n*-6 | 45.34 ± 0.20 |  | 39.78 ± 0.33^a^ |  | 41.47 ± 0.51^ab^ | 34.18 ± 0.36^abc^ |
| 20:2 *n*-6 | 0.11 ± 0.00 |  | - |  | 0.10 ± 0.00 | - |
| 20:3 *n*-6 | 0.12 ± 0.00 |  | - |  | 0.10 ± 0.00^ab^ | - |
| 20:4 *n*-6 | 0.22 ± 0.02 |  | 0.16 ± 0.01^a^ |  | 0.11 ± 0.01^a^ | 0.11 ± 0.01^a^ |
| Total | 45.92 ± 0.21 |  | 40.25 ± 0.31^a^ |  | 41.84 ± 0.49^ab^ | 34.49 ± 0.35^abc^ |
|  |  |  |  |  |  |  |
| *n-3 PUFA* |  |  |  |  |  |  |
| 18:3 *n*-3 | 0.81 ± 0.03 |  | 1.00 ± 0.03^a^ |  | 0.93 ± 0.05 | 0.91 ± 0.04 |
| 20:5 *n*-3 (EPA) | - |  | 0.57 ± 0.06^a^ |  | 0.18 ± 0.01^ab^ | 0.60 ± 0.04^ac^ |
| 22:5 *n*-3 | - |  | 0.33 ± 0.03^a^ |  | 0.10 ± 0.01^ab^ | 0.21 ± 0.01^abc^ |
| 22:6 *n*-3 (DHA) | - |  | 3.42 ± 0.35^a^ |  | 1.12 ± 0.08^ab^ | 3.63 ± 0.26^ac^ |
| Total | 0.91 ± 0.03 |  | 5.33 ± 0.43^a^ |  | 2.33 ± 0.09^ab^ | 5.34 ± 0.27^ac^ |
| Sum EPA+DHA | 0.08 ± 0.01 |  | 4.00 ± 0.40^a^ |  | 1.30 ± 0.09^ab^ | 4.22 ± 0.29^ac^ |
|  |  |  |  |  |  |  |

Fatty acid composition was analyzed in the total lipid fraction extracted from abdominal adipose tissue (epididymal fat depot). Results (mol %) are expressed as means ± SEM (*n*=7).

^a,b,c^Significant differences (ANOVA) compared with cHF, cHF+ω3TG, and cHF+ω3PL (10 g per kg diet), respectively. MUFA, monounsaturated fatty acids; PUFA, polyunsaturated fatty acids. – , ≤0.1 % (detection limit).
