# Supplementary material for: Metabolic Effects of n-3 PUFA as Phospholipids Are Superior to Triglycerides in Mice Fed a High-Fat Diet: Possible Role of Endocannabinoids
Source: PLoS One. 2012 Jun 11;7(6):e38834. doi: 10.1371/journal.pone.0038834 (PMC3372498; doi:10.1371/journal.pone.0038834)
Supplement: Table S8 — TOF-SIMS analysis of lipid fractions in the liver from the ‘prevention study’. Various lipid species were analyzed in the liver by the TOF-SIMS method. Data are expressed as the (cHF+ω3TG)/cHF and (cHF+ω3PL)/cHF normalised signal intensity ratios for lipid signals, originating from mice fed the control diet (cHF) and from mice fed the cHF-based experimental diets supplemented with the EPA and DHA concentrate either in the form of triglycerides (cHF+ω3TG) or marine phospholipids (cHF+ω3PL). DAG, diacylglycerol; PC, phosphatidylcholine; PE, phosphatidylethanolamine; PI, phosphatidylinositol. (DOC) [file pone.0038834.s011.doc]

**Table S8** TOF-SIMS analysis of lipid fractions in the liver from the ‘prevention study’

| Lipid type | Specific ion | cHF+ω3TG | cHF+ω3PL |
| --- | --- | --- | --- |
|  |  |  |  |
| Fatty acids | 16:2 | 1.05 | 1.01 |
|  | 16:1 | 1.17 | 1.06 |
|  | 16:0 | 1.14 | 1.15 |
|  | 18:3 | 1.05 | 0.81 |
|  | 18:2 | 1.04 | 0.89 |
|  | 18:1 | 1.02 | 0.94 |
|  | 18:0 | 0.85 | 1.00 |
|  | 20:5 | 1.75 | 1.96 |
|  | 20:4 | 0.31 | 0.37 |
|  | 20:3 | 0.60 | 0.75 |
|  | 20:2 | 0.66 | 0.66 |
|  | 22:6 | 2.42 | 2.41 |
|  | 22:5 | 1.42 | 1.47 |
| PI |  |  |  |
|  | 34:6 | 1.64 | 1.06 |
|  | 36:6 | 1.38 | 1.37 |
|  | 38:5 | 1.58 | 1.95 |
|  | 38:4 | 0.75 | 0.65 |
|  | 38:3 | 1.05 | 0.94 |
|  | 40:7 | 3.46 | 4.98 |
|  | 40:6 | 9.14 | 17.09 |
| PE |  |  |  |
|  | 34:3 | 1.45 | 0.91 |
|  | 34:2 | 1.27 | 1.06 |
|  | 36:4 | 0.87 | 0.60 |
|  | 36:3 | 1.26 | 0.93 |
|  | 36:2 | 1.18 | 1.09 |
|  | 38:6 | 1.12 | 1.61 |
|  | 38:5 | 1.15 | 1.48 |
|  | 38:4 | 0.40 | 0.39 |
|  | 40:8 | 1.23 | 0.90 |
|  | 40:7 | 1.55 | 1.40 |
|  | 40:6 | 1.84 | 2.24 |
|  | 42:10 | 0.59 | 0.43 |
| PC |  |  |  |
|  | 32:0 | 0.99 | 0.79 |
|  | 34:3 | 1.18 | 0.92 |
|  | 34:2 | 1.20 | 1.13 |
|  | 34:1 | 1.07 | 1.25 |
|  | 34:0 | 0.95 | 0.86 |
|  | 36:4 | 0.61 | 0.67 |
|  | 36:3 | 0.98 | 0.98 |
|  | 36:2 | 0.93 | 0.97 |
|  | 36:1 | 0.89 | 0.92 |
|  | 38:7 | 0.77 | 0.82 |
|  | 38:6 | 1.17 | 1.48 |
|  | 38:5 | 0.98 | 1.16 |
|  | 38:4 | 0.45 | 0.49 |
|  | 38:3 | 0.70 | 0.75 |
|  | 40:8 | 1.02 | 1.14 |
|  | 40:7 | 1.04 | 1.32 |
|  | 40:6 | 0.67 | 0.73 |
|  | 40:5 | 1.23 | 1.55 |
| DAG |  |  |  |
|  | 34:3 | 1.09 | 1.13 |
|  | 34:2 | 1.07 | 0.84 |
|  | 34:1 | 1.08 | 0.94 |
|  | 34:0 | 0.80 | 1.81 |
|  | 36:4 | 0.93 | 0.60 |
|  | 36:3 | 0.97 | 0.67 |
|  | 36:2 | 0.89 | 1.54 |
|  | 36:1 | 0.84 | 2.15 |
|  | 36:0 | 0.84 | 3.09 |

Various lipid species were analyzed in the liver by the TOF-SIMS method. Data are expressed as the (cHF+ω3TG)/cHF and (cHF+ω3PL)/cHF normalised signal intensity ratios for lipid signals, originating from mice fed the control diet (cHF) and from mice fed the cHF-based experimental diets supplemented with the EPA and DHA concentrate either in the form of triglycerides (cHF+ω3TG) or marine phospholipids (cHF+ω3PL).

DAG, diacylglycerol; PC, phosphatidylcholine; PE, phosphatidylethanolamine; PI, phosphatidylinositol.
