# Supplementary material for: Metabolic Effects of n-3 PUFA as Phospholipids Are Superior to Triglycerides in Mice Fed a High-Fat Diet: Possible Role of Endocannabinoids
Source: PLoS One. 2012 Jun 11;7(6):e38834. doi: 10.1371/journal.pone.0038834 (PMC3372498; doi:10.1371/journal.pone.0038834)
Supplement: Table S9 — TOF-SIMS analysis of lipid fractions in adipose tissue from the ‘prevention study’. Various lipid species were analyzed in epididymal adipose tissue by the TOF-SIMS method. Data are expressed as the (cHF+ω3TG)/cHF and (cHF+ω3PL)/cHF normalised signal intensity ratios for lipid signals, originating from mice fed the control diet (cHF) and from mice fed the cHF-based experimental diets supplemented with the EPA and DHA concentrate either in the form of triglycerides (cHF+ω3TG) or marine phospholipids (cHF+ω3PL). DAG, diacylglycerol; PE, phosphatidylethanolamine. (DOC) [file pone.0038834.s012.doc]

**Table S9** TOF-SIMS analysis of lipid fractions in adipose tissue from the ‘prevention study’

| Lipid type | Specific ion | cHF+ω3TG | cHF+ω3PL |
| --- | --- | --- | --- |
|  |  |  |  |
| Fatty acids | 16:2 | 1.12 | 1.10 |
|  | 16:1 | 1.19 | 1.23 |
|  | 16:0 | 1.39 | 1.56 |
|  | 18:3 | 0.87 | 0.80 |
|  | 18:2 | 0.87 | 0.77 |
|  | 18:1 | 0.89 | 0.92 |
|  | 18:0 | 1.30 | 1.39 |
|  | 20:5 | 3.75 | 3.91 |
|  | 20:4 | 0.84 | 0.72 |
|  | 20:3 | 0.87 | 0.80 |
|  | 20:2 | 0.90 | 0.92 |
|  | 22:6 | 12.65 | 14.98 |
|  | 22:5 | 4.11 | 3.21 |
| PE |  |  |  |
|  | 34:3 | 1.06 | 0.93 |
|  | 34:2 | 0.86 | 0.98 |
|  | 36:4 | 1.03 | 0.79 |
|  | 36:3 | 0.73 | 0.81 |
|  | 36:2 | 0.77 | 0.91 |
|  | 38:6 | 1.88 | 1.98 |
|  | 38:5 | 1.12 | 0.99 |
|  | 38:4 | 0.75 | 0.66 |
|  | 40:8 | 0.96 | 0.90 |
|  | 40:7 | 1.45 | 1.52 |
|  | 40:6 | 2.26 | 2.33 |
|  | 42:10 | 0.73 | 0.60 |
| DAG |  |  |  |
|  | 30:3 | 1.18 | 0.98 |
|  | 30:2 | 1.19 | 1.19 |
|  | 30:1 | 1.47 | 1.74 |
|  | 30:0 | 2.42 | 3.46 |
|  | 32:3 | 1.14 | 1.07 |
|  | 32:2 | 1.22 | 1.29 |
|  | 32:1 | 1.63 | 1.86 |
|  | 32:0 | 3.17 | 3.90 |
|  | 34:3 | 1.02 | 0.90 |
|  | 34:2 | 1.04 | 1.01 |
|  | 34:1 | 1.12 | 1.21 |
|  | 34:0 | 1.72 | 2.18 |
|  | 36:4 | 0.72 | 0.55 |
|  | 36:3 | 0.74 | 0.67 |
|  | 36:2 | 0.81 | 0.85 |
|  | 36:1 | 0.97 | 1.17 |
|  | 36:0 | 1.40 | 1.78 |
|  | 38:6 | 4.77 | 5.09 |
|  | 40:8 | 14.25 | 13.60 |
|  | 40:7 | 12.02 | 12.38 |
| Triacylglycerol |  |  |  |
|  | 48:4 | 0.87 | 1.13 |
|  | 48:3 | 1.11 | 1.14 |
|  | 48:2 | 1.42 | 1.49 |
|  | 48:1 | 1.64 | 1.76 |
|  | 48:0 | 2.56 | 3.06 |
|  | 50:5 | 1.34 | 1.06 |
|  | 50:4 | 1.01 | 0.93 |
|  | 50:3 | 1.07 | 1.16 |
|  | 50:2 | 1.28 | 1.41 |
|  | 50:1 | 1.44 | 1.73 |
|  | 52:5 | 0.94 | 0.78 |
|  | 52:4 | 0.83 | 0.75 |
|  | 52:3 | 0.90 | 0.95 |
|  | 52:2 | 1.05 | 1.19 |
|  | 54:7 | 1.36 | 1,23 |
|  | 54:6 | 0.75 | 0.60 |
|  | 54:5 | 0.64 | 0.54 |
|  | 54:4 | 0.70 | 0.69 |
|  | 54:3 | 0.88 | 1.03 |
|  | 56:9 | 1.89 | 1.65 |
|  | 56:8 | 3.86 | 3.76 |
|  | 56:7 | 3.09 | 3.04 |
|  | 58:11 | 1.26 | 1.08 |
|  | 58:10 | 2.48 | 2.04 |
|  | 58:9 | 4.23 | 3.67 |
|  | 58:8 | 3.43 | 3.43 |

Various lipid species were analyzed in epididymal adipose tissue by the TOF-SIMS method. Data are expressed as the (cHF+ω3TG)/cHF and (cHF+ω3PL)/cHF normalised signal intensity ratios for lipid signals, originating from mice fed the control diet (cHF) and from mice fed the cHF-based experimental diets supplemented with the EPA and DHA concentrate either in the form of triglycerides (cHF+ω3TG) or marine phospholipids (cHF+ω3PL). DAG, diacylglycerol; PE, phosphatidylethanolamine.
