# Supplementary material for: Metabolic Effects of n-3 PUFA as Phospholipids Are Superior to Triglycerides in Mice Fed a High-Fat Diet: Possible Role of Endocannabinoids
Source: PLoS One. 2012 Jun 11;7(6):e38834. doi: 10.1371/journal.pone.0038834 (PMC3372498; doi:10.1371/journal.pone.0038834)
Supplement: Table S10 — TOF-SIMS analysis of lipid fractions in the skeletal muscle from the ‘prevention study’. Various lipid species were analyzed in the skeletal muscle (m. quadriceps femoris) by the TOF-SIMS method. Data are expressed as the (cHF+ω3TG)/cHF and (cHF+ω3PL)/cHF normalised signal intensity ratios for lipid signals, originating from mice fed the control diet (cHF) and from mice fed the cHF-based experimental diets supplemented with the EPA and DHA concentrate either in the form of triglycerides (cHF+ω3TG) or marine phospholipids (cHF+ω3PL). DAG, diacylglycerol; PC, phosphatidylcholine; PE, phosphatidylethanolamine; PI, phosphatidylinositol. (DOC) [file pone.0038834.s013.doc]

**Table S10** TOF-SIMS analysis of lipid fractions in the skeletal muscle from the ‘prevention study’

| Lipid type | Specific ion | cHF+ω3TG | cHF+ω3PL |
| --- | --- | --- | --- |
|  |  |  |  |
| Fatty acids | 16:0 | 1.24 | 1.51 |
|  | 16:1 | 1.08 | 1.16 |
|  | 16:2 | 1.21 | 1.06 |
|  | 18:0 | 0.78 | 0.90 |
|  | 18:1 | 1.03 | 0.82 |
|  | 18:2 | 0.83 | 0.47 |
|  | 18:3 | 0.80 | 0.68 |
|  | 20:2 | 0.86 | 0.75 |
|  | 20:3 | 0.81 | 0.72 |
|  | 20:4 | 0.34 | 0.40 |
|  | 20:5 | 1.48 | 1.30 |
|  | 22:5 | 1.11 | 0.95 |
|  | 22:6 | 2.32 | 2.67 |
| PI |  |  |  |
|  | 36:6 | 0.90 | 0.49 |
|  | 38:4 | 0.55 | 0.51 |
|  | 40:6 | 5.06 | 6.12 |
| PE |  |  |  |
|  | 34:2 | 1.10 | 0.96 |
|  | 34:3 | 1.32 | 1.14 |
|  | 36:2 | 0.70 | 0.57 |
|  | 36:3 | 1.16 | 0.88 |
|  | 36:4 | 0.97 | 0.83 |
|  | 38:4 | 0.60 | 0.48 |
|  | 38:5 | 1.00 | 0.92 |
|  | 38:6 | 1.09 | 1.59 |
|  | 40:6 | 1.07 | 1.21 |
|  | 40:7 | 1.08 | 1.20 |
|  | 40:8 | 1.09 | 1.06 |
|  | 42:10 | 1.19 | 0.87 |
| PC |  |  |  |
|  | 32:0 | 1.27 | 1.29 |
|  | 32:1 | 0.85 | 0.93 |
|  | 34:0 | 0.82 | 0.77 |
|  | 34:1 | 0.59 | 0.69 |
|  | 34:2 | 0.49 | 0.43 |
|  | 34:3 | 1.06 | 1.00 |
|  | 36:2 | 1.01 | 0.98 |
|  | 36:3 | 1.05 | 1.18 |
|  | 36:4 | 0.39 | 0.40 |
|  | 38:5 | 0.95 | 1.12 |
|  | 38:6 | 2.12 | 2.85 |
|  | 38:7 | 0.87 | 0.88 |
|  | 40:7 | 1.33 | 1.26 |
| DAG |  |  |  |
|  | 30:0 | 0.81 | 1.28 |
|  | 30:1 | 0.66 | 1.05 |
|  | 30:2 | 0.81 | 1.31 |
|  | 30:3 | 0.67 | 1.30 |
|  | 32:0 | 0.98 | 1.90 |
|  | 32:1 | 1.08 | 1.26 |
|  | 32:2 | 0.95 | 1.11 |
|  | 32:3 | 0.69 | 0.95 |
|  | 34:0 | 0.98 | 1.33 |
|  | 34:1 | 1.24 | 1.02 |
|  | 34:2 | 1.08 | 0.84 |
|  | 34:3 | 0.97 | 0.85 |
|  | 36:0 | 0.78 | 1.27 |
|  | 36:1 | 0.94 | 1.03 |
|  | 36:2 | 0.94 | 0.69 |
|  | 36:3 | 0.99 | 0.62 |
|  | 36:4 | 0.99 | 0.55 |
|  | 38:6 | 1.56 | 2.16 |
|  | 40:7 | 1.70 | 2.01 |
|  | 40:8 | 1.73 | 1.66 |

Various lipid species were analyzed in the skeletal muscle (*m. quadriceps femoris*) by the TOF-SIMS method. Data are expressed as the (cHF+ω3TG)/cHF and (cHF+ω3PL)/cHF normalised signal intensity ratios for lipid signals, originating from mice fed the control diet (cHF) and from mice fed the cHF-based experimental diets supplemented with the EPA and DHA concentrate either in the form of triglycerides (cHF+ω3TG) or marine phospholipids (cHF+ω3PL). DAG, diacylglycerol; PC, phosphatidylcholine; PE, phosphatidylethanolamine; PI, phosphatidylinositol.
