# Supplementary material for: Metabolic Effects of n-3 PUFA as Phospholipids Are Superior to Triglycerides in Mice Fed a High-Fat Diet: Possible Role of Endocannabinoids
Source: PLoS One. 2012 Jun 11;7(6):e38834. doi: 10.1371/journal.pone.0038834 (PMC3372498; doi:10.1371/journal.pone.0038834)
Supplement: Table S11 — The levels of endocannabinoids and endocannabinoid-like molecules in plasma and epididymal adipose tissue of dietary obese mice from the ‘reversal study’. To induce obesity, dyslipidemia and glucose intolerance, mice were fed the cHF diet between 3 and 7 months of age, and then for 9 more weeks by different diets (all containing 2 g metformin per kg diet). cHF+ω3TG and cHF+ω3PL diets were supplemented with ∼30 g DHA/EPA per kg diet. Data (ng/g tissue) are expressed as means±SEM (plasma, n = 2 of pooled samples from 3 mice; adipose tissue, n = 8−9). aSignificant differences compared with cHF (t-test or ANOVA). bSignificant differences compared with cHF+ω3TG (ANOVA). 2-AG, 2-arachidonoylglycerol; AEA, anandamide; DHEA, N-docosahexaenoylethanolamine; EPEA, N-eicosapentaenoylethanolamine; OEA, oleoylethanolamine; PEA, palmitoylethanolamine; SEA, stearoylethanolamine; DLE, dihomo-γ-linolenoylethanolamine. (DOC) [file pone.0038834.s014.doc]

**Table S11** The levels of endocannabinoids and endocannabinoid-like molecules in plasma and epididymal adipose tissue of dietary obese mice from the ‘reversal study’

|  | cHF | cHF+ω3TG | cHF+ω3PL |
| --- | --- | --- | --- |
| *Plasma* |  |  |  |
| 2-AG | 56 ± 8 | 23 ± 2 | 19 ± 1 |
| AEA | 0.48 ± 0.02 | 0.13 ± 0.01 | 0.09 ± 0.01 |
| DHEA | 0.81 ± 0.01 | 2.55 ± 0.04 | 5.15 ± 0.05 |
| EPEA | 0.00 ± 0.00 | 0.23 ± 0.06 | 0.49 ± 0.09 |
| DLE | 0.11 ± 0.01 | 0.06 ± 0.00 | 0.04 ± 0.01 |
| OEA | 5.50 ± 0.29 | 3.86 ± 0.16 | 4.96 ± 0.10 |
| PEA | 4.28 ± 0.06 | 4.68 ± 0.10 | 5.80 ± 0.22 |
| SEA | 3.46 ± 0.04 | 2.76 ± 0.03 | 3.28 ± 0.19 |
| *Adipose tissue* |  |  |  |
| 2-AG | 187 ± 36 | 79 ± 13^a^ | 46 ± 4^a^ |
| AEA | 2.68 ± 0.12 | 1.08 ± 0.07^a^ | 1.08 ± 0.07^a^ |
| DHEA | 2.40 ± 0.15 | 14.92 ± 1.63^a^ | 31.37 ± 7.26^ab^ |
| EPEA | 0.00 ± 0.00 | 3.96 ± 0.55^a^ | 6.33 ± 1.00^a^ |
| DLE | 0.36 ± 0.02 | 0.24 ± 0.01^a^ | 0.22 ± 0.03^a^ |
| OEA | 43 ± 3 | 48 ± 4 | 46 ± 5 |
| PEA | 20 ± 1 | 26 ± 2 | 25 ± 3 |
| SEA | 44 ± 2 | 52 ± 3 | 51 ± 4 |
|  |  |  |  |

To induce obesity, dyslipidemia and glucose intolerance, mice were fed the cHF diet between 3 and 7 months of age, and then for 9 more weeks by different diets (all containing 2 g metformin per kg diet). cHF+ω3TG and cHF+ω3PL diets were supplemented with ~30 g DHA/EPA per kg diet. Data (ng/g tissue) are expressed as means±SEM (Plasma, *n*=2 of pooled samples from 3 mice; adipose tissue, *n*=8-9). ^a^Significant differences compared with cHF (t-test or ANOVA). ^b^Significant differences compared with cHF+ω3TG (ANOVA). 2-AG, 2-arachidonoylglycerol; AEA, anandamide; DHEA, *N*-docosahexaenoylethanolamine; EPEA, *N*-eicosapentaenoylethanolamine; OEA, oleoylethanolamine; PEA, palmitoylethanolamine; SEA, stearoylethanolamine; DLE, dihomo-γ-linolenoylethanolamine.
